# Stress Reactivity, Susceptibility to Hypertension, and Differential Expression of Genes in Hypertensive Compared to Normotensive Patients

**DOI:** 10.3390/ijms23052835

**Published:** 2022-03-04

**Authors:** Dmitry Oshchepkov, Irina Chadaeva, Rimma Kozhemyakina, Karina Zolotareva, Bato Khandaev, Ekaterina Sharypova, Petr Ponomarenko, Anton Bogomolov, Natalya V. Klimova, Svetlana Shikhevich, Olga Redina, Nataliya G. Kolosova, Maria Nazarenko, Nikolay A. Kolchanov, Arcady Markel, Mikhail Ponomarenko

**Affiliations:** 1 Institute of Cytology and Genetics, Siberian Branch of Russian Academy of Sciences, 630090 Novosibirsk, Russia; diman@bionet.nsc.ru (D.O.); ichadaeva@bionet.nsc.ru (I.C.); korimma@bionet.nsc.ru (R.K.); ka125699ri@yandex.ru (K.Z.); b.khandaev@g.nsu.ru (B.K.); sharypova@bionet.nsc.ru (E.S.); pon.petr@gmail.com (P.P.); mantis_anton@bionet.nsc.ru (A.B.); klimova@bionet.nsc.ru (N.V.K.); shikhsvt@bionet.nsc.ru (S.S.); oredina@bionet.nsc.ru (O.R.); kolosova@bionet.nsc.ru (N.G.K.); kol@bionet.nsc.ru (N.A.K.); markel@bionet.nsc.ru (A.M.); 2 Institute of Medical Genetics, Tomsk National Research Medical Center, 634009 Tomsk, Russia; maria-nazarenko@medgenetics.ru

**Keywords:** human, hypertension, stress reactivity, molecular marker, *Rattus norvegicus*, RNA-Seq, qPCR, differentially expressed gene, meta-analysis, correlation, principal component, bootstrap

## Abstract

Although half of hypertensive patients have hypertensive parents, known hypertension-related human loci identified by genome-wide analysis explain only 3% of hypertension heredity. Therefore, mainstream transcriptome profiling of hypertensive subjects addresses differentially expressed genes (DEGs) specific to gender, age, and comorbidities in accordance with predictive preventive personalized participatory medicine treating patients according to their symptoms, individual lifestyle, and genetic background. Within this mainstream paradigm, here, we determined whether, among the known hypertension-related DEGs that we could find, there is any genome-wide hypertension theranostic molecular marker applicable to everyone, everywhere, anytime. Therefore, we sequenced the hippocampal transcriptome of tame and aggressive rats, corresponding to low and high stress reactivity, an increase of which raises hypertensive risk; we identified stress-reactivity-related rat DEGs and compared them with their known homologous hypertension-related animal DEGs. This yielded significant correlations between stress reactivity-related and hypertension-related fold changes (log2 values) of these DEG homologs. We found principal components, PC1 and PC2, corresponding to a half-difference and half-sum of these log2 values. Using the DEGs of hypertensive versus normotensive patients (as the control), we verified the correlations and principal components. This analysis highlighted downregulation of β-protocadherins and hemoglobin as whole-genome hypertension theranostic molecular markers associated with a wide vascular inner diameter and low blood viscosity, respectively.

## 1. Introduction

Hypertension is a fatal yet preventable risk factor of ischemic heart disease [1], the top cause of death worldwide [2]. Besides essential hypertension, there are many cases of hypertension that are not clinically classified as essential. In all these cases, there is an increase in intravascular pressure (only local sometimes) together with vascular shear stress, oxidative stress, inflammatory reactions, and remodeling of the vascular wall. These pathogenic mechanisms common to all hypertensive conditions share, at least in part, a molecular basis that we are trying to pinpoint here.

Although the hypertensive risk increases with age [3,4], the age of the first clinical manifestations of hypertension is now diminishing [5]. Preeclampsia (hypertension in pregnancy) is becoming a challenge to obstetricians [6]. Prenatal stress can epigenetically reprogram a newborn’s development and can lead to clinical hypertension in adulthood [7]. Pulmonary hypertension may start to develop in newborns [8]. Hypertension co-occurs with cancer [9,10,11,12,13], cirrhosis [14], and prostatitis [15]. Hypertension worsens both injury [16] and transplantation [17] of a kidney. Epilepsy [18], psoriasis, and dermatitis [19] are associated with hypertension. Anti–SARS-CoV-2 antibody titers are lower in hypertensive than in normotensive patients [20]. The “mosaic theory” of hypertension [21] was recently enriched with hypertensive development via exosome-dependent inflammation and angiogenesis impairment, associated with endothelial dysfunction and vascular remodeling [22]. A clinical review [23] revealed that the hypertensive risk increases with an increase in patients’ stress reactivity [24]. Parents of half of the hypertensive patients had hypertension, but all the known (>60) hypertension-related whole-genome human loci explained only 3% of this heritability of hypertension [25]. Maybe this is why mainstream transcriptome-profiling studies on hypertensive versus normotensive patients [26,27,28,29,30,31,32,33,34,35,36,37,38,39] and animals [7,40,41,42,43,44,45,46,47,48,49,50,51,52,53,54,55,56,57] are focused on the differentially expressed genes (DEGs) that are specific to gender, age, and the stage of hypertension development. This is needed in predictive preventive personalized participatory (4P) medicine [58] to estimate where, how, why, and when hypertension might occur in a given patient depending on his/her genetic background. Because hypertension seems to have a finger in every pie, a meta-analysis of all the available specific hypertension-related DEGs can find among them a theranostic molecular marker of hypertension applicable to everyone, everywhere, anytime.

In our previous studies within this mainstream paradigm, we measured stress reactivity in rats [59] and created an inbred ISIAH rat strain (i.e., inherited stress-induced arterial hypertension) [60] and two outbred strains—tame and aggressive rats—corresponding to low and high stress reactivity [61,62,63,64]. On this basis, we sequenced transcriptomes in the brain stem [43], hypothalamus [44], renal medulla [45], renal cortex [46], and adrenal glands [47] of hypertensive ISIAH rats versus normotensive WAG rats. Besides this, we profiled transcriptomes of the hippocampus [40], prefrontal cortex [41], and retina [42] in OXYS rats (ICG SB RAS, Novosibirsk, Russia), which spontaneously develop the accelerated-senescence phenotype against a background of moderately high blood pressure [65,66,67,68] with respect to normotensive Wistar rats. In the present work, we meta-analyzed our eight abovementioned RNA-Seq datasets to ensure out of caution that among them (together with those available in PubMed [69]), there are still no invariant molecular markers of hypertension. Accordingly, we sequenced the hippocampal transcriptome of tame compared to aggressive rats and identified the stress-reactivity-related rat DEGs and—using our bioinformatics model [70,71,72]—compared them by homology with all the available hypertension-related animal DEGs that we could find. The results were verified using the DEGs of hypertensive versus normotensive patients.

## 2. Results

### 2.1. RNA-Seq and Mapping to the Reference Rat Genome

We sequenced the hippocampal transcriptome of three adult male tame gray rats (*Rattus norvegicus*)—in comparison with that of three aggressive ones—on an Illumina NextSeq 550 system (see Section 4.2). We chose the hippocampus because its functions contribute to learning under stress [73]. The rats were derived from two outbred tame and aggressive strains selectively bred at the ICG SB RAS [59,64] for over 90 generations using the glove test as described elsewhere [74]. The rats were not consanguineous (see Section 4.1). This procedure yielded 169,529,658 raw reads of 75 nt in length (Table 1); we deposited them in the NCBI SRA database [75] (ID PRJNA668014).

In Table 1, the reader can see that 146,521,467 reads could be aligned with rat reference genome Rn6 and yielded 14,039 genes expressed within the hippocampus of the rats under study. Using Fisher’s Z-test with Benjamini’s correction for multiple comparisons, we found 42 DEGs that were not hypothetical, tentative, predicted, uncharacterized, or protein-non-coding genes; this approach reduced the false-positive error rates (Table 1 and Table 2).

### 2.2. Quantitative PCR (qPCR)-Based Selective Verification of the DEGs Identified in this Work in the Hippocampus of Tame versus Aggressive Rats

**Figure 1:**
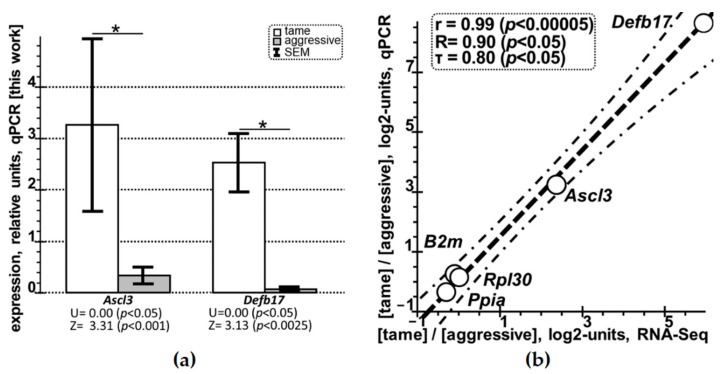
qPCR-based selective verification of the DEGs identified by RNA-Seq in this work in the hippocampus of tame versus aggressive rats. *Legend*: (**a**) in tame male adult rats (white bars) versus aggressive ones (grey bars), both DEGs examined (i.e., *Ascl3* and *Defb17*) are statistically significantly overexpressed in the hippocampus (here, bar height (i.e., mean), error bars (i.e., standard error of the mean [SEM]), and asterisks denote statistical significance at *p* < 0.05 according to both the nonparametric Mann–Whitney *U*-test and parametric Fisher’s *Z*-test). Asterisk (symbol “*”), statistically significant at *p* < 0.05. (**b**) Statistically significant correlations between the relative expression levels of the two selected DEGs and three reference genes (i.e., *B2m* (β-2-microglobulin), *Ppia* (peptidylprolyl isomerase A), and *Rpl30* (ribosomal protein L30)) in the hippocampus of tame versus aggressive rats (open circles), as measured experimentally by RNA-Seq (X-axis) and qPCR (Y-axis) and presented on the log2 scale (see “Materials and Methods”). Dashed and dash-and-dot lines denote linear regression and boundaries of its 95% confidence interval calculated using Statistica software (Statsoft^TM^, Tulsa, OK, USA). r, R, τ, and *p* are coefficients of Pearson’s linear correlation, Spearman’s rank correlation, Kendall’s rank correlation, and their *p* values (statistical significance), respectively.

First, we used 16 additional unrelated rats, namely: eight aggressive and eight tame rats that scored “–3” and “3”, respectively, on a scale from –4 (most aggressive rat) to 4 (tamest rat) in the glove test [74] conducted one month before the extraction of hippocampus samples (Table 3). Next, among the 42 DEGs listed in Table 2, we chose *Ascl3* and *Defb17*; our qPCR data on them in the hippocampus of the tame and aggressive rats (see Section 4.4) are in Table 3 as the “mean ± standard error of the mean” (M_0_ ± SEM) of their expression relative to four reference genes (*B2m*, *Hprt1*, *Ppia*, and *Rpl30*) [76] in triplicate. Arithmetic-mean estimates of the expression levels of each gene (*Ascl3* and *Defb17)* in the hippocampus of these tame and aggressive rats in question are given in Table 3 and Figure 1a.

According to both the Mann–Whitney *U* test and Fisher’s Z-test, both *Ascl3* and *Defb17* are significantly overexpressed in the hippocampus of the tame (white bars) versus aggressive (grey bars) rats according to the qPCR data obtained here (Figure 1a: *p* < 0.05, asterisks), consistently with the RNA-Seq data (Table 2). Figure 1b depicts a significant Pearson’s linear correlation (*p* < 0.00005), Spearman’s rank correlation (*p* < 0.05), and Kendall’s rank correlation (*p* < 0.05) between the log2 values (hereinafter, log2: the log2-transformed ratio of an expression level of a given gene in tame rats to that in aggressive rats) for five genes—*Ascl3*, *Defb17*, *B2m*, *Ppia*, and *Rpl30* (open circles)—within the RNA-Seq (X-axis) and qPCR (Y-axis) data obtained here.

### 2.3. Comparison of the Known DEGs (of Hypertensive versus Normotensive Animals) with Their Homologous Genes among the 42 Hippocampal DEGs (of Tame versus Aggressive Rats) Identified Here

In this study, using the PubMed database [69], we compiled all the transcriptomes (that we could find) of hypertensive versus normotensive animals, as presented in Table 4. The total number of DEGs was 4216 in 14 tissues of four animal species, as cited in the rightmost column of Table 4 [7,40,41,42,43,44,45,46,47,48,49,50,51,52,53,54,55,56,57].

Figure S1 (hereinafter: see Supplementary Materials) depicts how we compared 4216 DEGs of hypertensive versus normotensive animals (Table 4) with 42 hippocampal DEGs of the tame versus aggressive rats (Table 2). First, we compiled 151 pairs of homologous DEGs, where one DEG was taken from Table 2, while its homologous DEG was found among the 4216 DEGs described in Table 4 (both are in Table S1 (hereinafter: see Supplementary Materials)), as shown in Figure S1 using a Venn diagram and in the table. Next, for the first time. we found that stress-reactivity-related and hypertension-related log2 values of the homologous animal DEGs statistically significantly correlate with each other according to Pearson’s linear correlation (r = −0.29, *p* < 0.0005), the Goodman–Kruskal generalized correlation (γ = −0.20, *p* < 0.0005), and Spearman’s (R = −0.29, *p* < 0.00025) and Kendall’s (τ = −0.20, *p* < 0.0005) rank correlations. Finally, we processed Table S1 by principal component analysis in the Bootstrap mode of the PAST4.04 software [77] that yielded principal components PC1 and PC2, corresponding to a half-difference and half-sum of the stress reactivity-related and hypertension-related log2 values of the homologous animal DEGs (Figure S1).

### 2.4. Verification of the Results Obtained on the Hypertensive versus Normotensive Animals Examined in this Work with respect to the DEGs—Of Hypertensive versus Normotensive Patients—That We Could Find

Using the PubMed database [69], we collected all the DEGs (of hypertensive compared with normotensive patients) that we could find (Table 5). The total number of hypertension-related human DEGs found was 7865, as cited in the rightmost column of Table 5 [26,27,28,29,30,31,32,33,34,35,36,37,38,39].

**Figure 2:**
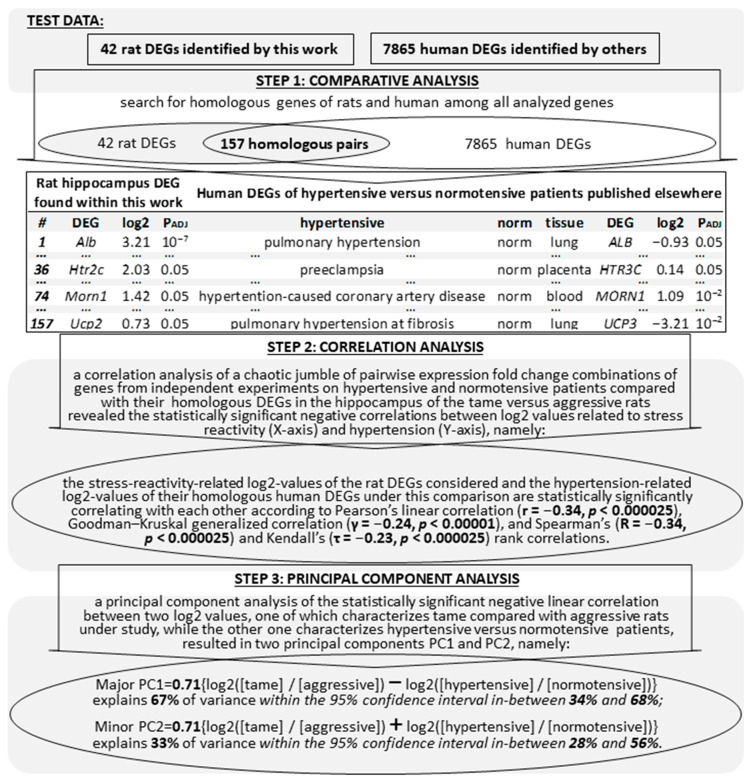
A step-by-step diagram of verification of the obtained results on the hypertensive versus normotensive animals examined in this work with respect to all the transcriptomes (that we could find) of hypertensive versus normotensive patients (Table 5). *Legend*: see the footnote of Table 2; PC1 and PC2: principal components calculated using the PAST4.04 software [77].

Figure 2 shows exactly how we reproduced step-by-step the results obtained from the hypertension-related animal DEGs only by replacing them with the hypertension-related human DEGs (Table 5) as independent control clinical data that are documented in Table S2. The lower half of this figure presents robust correlations between the stress-reactivity-related and hypertension-related log2 values corresponding to animal and human DEG homologs as well as principal components PC1 and PC2 proportional to the half-difference and half-sum, respectively, of these log2 values; this was the essence of the verification.

### 2.5. Searching for the Hypertension-Related Molecular Markers among the Human Genes Orthologous to the 42 Hippocampal DEGs (of Tame versus Aggressive Rats) Identified in this Work

To this end, first of all, using the PubMed database [69], we characterized each of the 42 hippocampal DEGs (of tame versus aggressive rats) identified in this work (Table 2), in terms of how downregulation or upregulation of their orthologous human genes can manifest itself in hypertension, as presented [78,79,80,81,82,83,84,85,86,87,88,89,90,91,92,93,94,95,96,97,98,99,100,101,102,103,104,105,106,107,108,109,110,111,112,113,114,115,116,117,118,119,120,121,122,123,124,125,126,127,128,129,130,131,132,133,134,135,136,137,138,139,140,141,142,143,144,145,146,147,148,149,150,151,152,153,154,155,156,157,158,159,160,161,162,163,164,165,166,167,168,169,170,171,172,173,174,175,176,177,178,179,180,181,182,183,184,185,186] in Table S3 (hereinafter: see Supplementary Materials). 

Next, for each hippocampal DEG (of tame versus aggressive rats) in question (Table 2), we determined how many homologous DEGs of hypertensive versus normotensive subjects (i.e., patients and animals) have the opposite (N_PC1_) or the same (N_PC2_) sign of their log2 values related to hypertension, as compared with the sign of the log2 value of this hippocampal DEG in tame versus aggressive rats, because principal components PC1 and PC2 correspond to a half-difference and half-sum, respectively, of these log2 values (Figure S1 and Figure 2). 

Table 6 presents these determined quantities (N_PC1_ and N_PC2_) together with their statistical significance assessed via the binomial distribution both without (*p*-values) and with (*P_ADJ_*-values) Bonferroni’s correction for multiple comparisons. 

As shown in this table, only two of the 42 DEGs (in the hippocampus of the tame versus aggressive rats) found here are linked with PC1 (i.e., *Hbb-b1* and *Pcdhb9*, as described in Table 7). Looking through Table 7, readers can see the statistically significant upregulation of both β-protocadherin and hemoglobin subunit DEGs in the tissues of the hypertensive versus normotensive subjects (patients and animals). This result allowed us to propose the statistically significant downregulation of their homologous DEGs (in the hippocampus of tame versus aggressive rats), identified here (Table 2) as candidate hypertension theranostic molecular markers. 

### 2.6. Verification of Downregulation of Human β-Hemoglobin and β-Protocadherins as HypertensionTtheranostic Molecular Markers using the DEGs (That We Could Find) of Domestic versus Wild Animals 

For this purpose, using the PubMed database [69], we collected all the transcriptomes (that we could find) of domestic animals compared with their wild congeners, as shown in Table 8. The bottom row of this table indicates that we found 2393 DEGs in the tissues of domestic versus wild animals, as cited in the rightmost column of this table [72,187,188,189,190,191,192,193].

Using the 42 DEGs (from the hippocampus of the tame versus aggressive rats) identified here (Table 2), together with these 2393 DEGs of domestic versus wild animals (Table 8), we revealed three β-protocadherin DEGs and seven hemoglobin subunit DEGs, which are compared in Table 9 with the human homologous genes (*HBB*, *HBD*, and *PCDHB9*)*,* annotated with respect to hypertension in Table S3. Within columns viii and ix of this table, we transformed the log2 value characterizing the animal hemoglobin subunit and β-protocadherin DEGs into either underexpression or overexpression of the corresponding gene during divergence of domestic and wild animals from their most recent common ancestor, which is the most widely used phylogeny concept [194,195,196,197,198]. Downregulation of human genes *HBB* and *HBD* reduces blood viscosity [199] and corresponds to downregulation of the homologous genes *Hbb-b1*, *Hbbl*, *Hba1*, *Hbad*, *Hbm,* and *Hbz1* in the tame rat, domestic chicken, or dog during their divergence from their most recent ancestors with respect to their wild congeners (Table 9). As for human hemoglobin upregulation, a high-altitude environment provokes both hypertension and hyperhemoglobinemia [103]. This hemoglobin upregulation in humans corresponds to high hemoglobin subunit levels in aggressive rats [72], wolves [189], and wild chickens [193] during their microevolution (Table 9). Likewise, human gene *PCDHB9* (protocadherin β9) downregulation leads to a wide vascular inner diameter [200] and corresponds to downregulation of β-protocadherins in tame rats [72] and domestic rabbits [188] during their microevolution (Table 9). Finally, PCDHB9 upregulation in humans elevates the risk of gastric cancer [201] (the surgical removal of which leads to hypertensive remission [12]) and corresponds to upregulation of β-protocadherins in aggressive rats [72] and wild rabbits [188] during their microevolution (Table 9). As a standard Fisher’s 2 × 2 table, Table 10 summarizes the observations detailed in Table 9.

As one can see in Table 10, downregulation of the genes of β-protocadherins and hemoglobin subunits, which were associated with a wide vascular inner diameter [200] and low blood viscosity [199], respectively, was observed only in domestic animals (not in their wild congeners). This difference is statistically significant according to the binomial distribution (*p* < 0.0001), Pearson’s χ^2^ test (*p* < 0.001), and Fisher’s exact test (*p* < 0.001). Thus, downregulation of β-protocadherins and downregulation of hemoglobin subunits in animals are molecular markers of low stress reactivity [24], which is both a key physiological trait for domestic animals [61,62] and a clinically proven hypertension theranostic physiological marker in everyone, everywhere, anytime [23].

## 3. Discussion

Here, we observed for the first time that downregulation of hemoglobin subunits or β-protocadherins corresponds to low blood viscosity or a wide vascular inner diameter, i.e., two universal genome-wide hypertension theranostic molecular markers applicable to everyone, everywhere, anytime, as readers can see in Table 7. Because of atherosclerosis comorbid with hypertension, this may support our previous finding that natural selection against underexpression of atheroprotective genes slows atherogenesis [202].

Nevertheless, it seems to be highly debatable how low expression levels of human genes *HBB*, *HBD*, and *PCDHB9* would be adaptive under natural selection, favoring their downregulation that could cause their loss. For this reason, here, we analyzed these genes using our web service SNP_TATA_Comparator [203] applicable to research on hypertension, owing to its successful use in a clinical study on pulmonary tuberculosis [204] comorbid with hypertension [205]. Figure S2 exemplifies how we also used the UCSC Browser [206], Bioperl toolkit [207], and a package of R [208], together with both Ensembl [209] and dbSNP [210] databases in the case of the candidate SNP marker (rs34166473) reducing blood viscosity via *HBD* downregulation [199], as outlined here (Table S4). In total, we examined all 85 SNPs within the 70 bp proximal promoters of the genes *HBD*, *HBD*, and *PCDHB9* within build #153 of the dbSNP database [210]. As a result of this work, we found 27 candidate SNP markers of hypertension, as indicated [12,103,199,200,201,211] in Table S4 and described [212,213,214,215,216,217,218,219,220] in Section S1 “Supplementary methods for DNA sequence analysis” (see Supplementary Materials).

Besides this, Figure S3 (hereinafter: see Supplementary Materials) presents the selective experimental verification [221,222,223] of these estimates (in an electrophoretic mobility shift assay; EMSA) exemplified by minor allele −30C of rs1473693473 (see Section S2 “Supplementary methods for in vitro measurements”). In total, we verified two ancestral alleles of the human *HBB* and *HBD* genes along with nine minor alleles, namely: rs35518301:g, rs34166473:c, rs34500389:t, rs33980857:a, rs34598529:g, rs33931746:g, rs33931746:c, rs281864525:c, and rs63750953:deletion (Table S5 (hereinafter: see Supplementary Materials)). According to Goodman–Kruskal generalized correlation (γ), Pearson’s linear correlation (r), and Spearman’s (R) and Kendall’s (τ) rank correlations, our computational predictions and experimental measurements are in significant agreement with one another (Figure S3c). 

Finally, according to the semicentennial tradition, to assess the relative mutation rates (e.g., transitions versus transversions [224], synonymous versus non-synonymous substitutions [225], and insertions versus deletions [226]), we compared the genes *HBB*, *HBD,* and *PCDHB9* in question with the human genome as a whole [227,228,229] (Table 11). 

At the top of this table is a genome-wide SNP pattern of TBP sites—where SNPs decreasing the TBP–DNA affinity dominate over SNPs, thus increasing this affinity within the human genome—as predicted by taking into account many mutagenesis molecular mechanisms (e.g., epistatic effects) [227] and as proven within the “1000 Genomes” project [228]. In accordance with Haldane’s dilemma [229] and neutral evolution theory [230], this whole-genome trait reflects neutral mutation drift as a norm. At the bottom of Table 11 is the hypertension-related candidate SNP markers identified here, which often significantly reduce the affinity of TBP for promoters of the genes *HBB*, *HBD*, and *PCDHB9*, representing the genome-wide neutral mutational drift antagonizing hypertension. 

Altogether, the hypertension-related candidate SNP markers discussed above fit the newest concept [231]: in addition to the accumulation of degenerative SNPs owing to their uncontrollability during neutral mutational drift, some adaptive SNPs can also accumulate in this way (Table 11).

## 4. Materials and Methods 

### 4.1. Animals

The study was conducted on adult male gray rats (*R. norvegicus*) artificially bred for over 90 generations for either aggressive or tame behavior (as two outbred strains). The rats were kept under standard conditions of the Conventional Animal Facility at the ICG SB RAS (Novosibirsk, Russia), as described elsewhere [64,74,232]. The total number of rats was 22 (11 aggressive and 11 tame ones), each four months old and weighing 250–270 g, all from different unrelated litters. All the rats were decapitated. Using a handbook technique [233], we excised samples of the hippocampus, which were then flash-frozen in liquid nitrogen and stored at −70 °C until use. Every effort was made to minimize the number of animals under study and to prevent their suffering. This work was conducted in accordance with the guidelines of the Declaration of Helsinki, Directive 2010/63/EU of the European Parliament, and of the European Council resolution of 22 September 2010. 

The research protocol was approved by the Interinstitutional Commission on Bioethics at the ICG SB RAS, Novosibirsk, Russia (approval documentation no. 8 dated 19 March 2012).

### 4.2. RNA-Seq

Total RNA was isolated from ~100 mg of the hippocampus tissue samples of tame (*n* = 3) and aggressive (*n* = 3) rats using the TRIzol™ reagent (Invitrogen, Carlsbad, CA, USA). The quality of the total-RNA samples was evaluated using a Bioanalyzer 2100 (Agilent, Santa-Clara, CA, USA). Samples with optimal RNA Integrity Numbers (RINs) were chosen for further analysis. Additionally, the total RNA was analyzed quantitatively on an Invitrogen Qubit™ 2.0 fluorometer (Invitrogen). Different RNA types were separated with the mirVana™ Kit (Thermo Fisher Scientific, Waltham, MA, USA). The Dynabeads mRNA Purification Kit (Invitrogen) was used to prepare highly purified mRNA from 5 μg of the RNA fraction depleted of small RNAs. Preparation of RNA-seq libraries from 15–30 ng of an mRNA fraction was carried out using the ScriptSeq™ v2 RNA-Seq Library Preparation Kit (epicenter^®^, Madison, WI, USA). The quality of the obtained libraries was checked on a Bioanalyzer 2100. After normalization, barcoded libraries were pooled and handed over to the Multi-Access Center of Genomic Research (ICG SB RAS, Novosibirsk, Russia) for sequencing on an Illumina NextSeq 550 instrument in a NextSeq^®^ 500/550 High Output Kit v2 cassette (75 cycles) under the assumption of a direct read of 75 nucleotides, with at least 40 million reads.

### 4.3. Mapping of RNA Sequences to the R. norvegicus Reference Genome

First, the primary raw Fastq files were checked by means of a quality control tool FastQC (https://www.bioinformatics.babraham.ac.uk/projects/fastqc; accessed on 19 December 2018) for high-throughput sequencing data. After that, using the Trimmomatic tool [234], we improved the quality of the raw reads step-by-step as follows: (i) removing a base from either the start or end position if the quality was low, (ii) trimming bases by a sliding-window method, and (iii) removing any remaining reads that were less than 36 bases long. Next, with the help of the TopHat2 toolbox [235], we aligned the trimmed reads to the *R. norvegicus* reference genome (RGSC Rnor_6.0, UCSC version Rn6, July 2014 assembly). Then, in SAMTools version 1.4 [236], we reformatted these alignments into sorted BAM files. After that, using the htseq-count tool from preprocessing software HTSeq v.0.7.2 [237], along with gtf files carrying coordinates of the rat genes according to Rnor_6.0 and an indexed SAM file, we assigned the reads in question to these genes. Finally, in DESeq2 [238] via Web service IRIS (http://bmbl.sdstate.edu/IRIS/; accessed on 16 January 2020), we rated the differential expression of the abovementioned rat genes, and to minimize false-positive error rates, applied Fisher’s Z-test [239] with Benjamini’s correction for multiple comparisons, as well as discarded all the hypothetical, tentative, predicted, uncharacterized, and protein-non-coding genes.

### 4.4. qPCR

To selectively and independently verify the tame-versus-aggressive rat hippocampal DEGs found here (Table 2), in this work, we performed a qPCR control assay on the total RNA taken only from the remaining samples of the hypothalamus of tame (*n* = 8) and aggressive (*n* = 8) rats. First, with the help of TRIzol™, we isolated total RNA, purified it on Agencourt RNAClean XP Kit magnetic beads (Beckman, #A63987), and quantified it by means of a Qubit™ 2.0 fluorometer (Invitrogen/Life Technologies) along with an RNA High-Sensitivity Kit (Invitrogen, cat. # Q32852). After that, we synthesized cDNA using the Reverse Transcription Kit (Syntol, #OT-1). Next, using web service PrimerBLAST [240], we designed oligonucleotide primers for qPCR (Table 12). 

After that, we carried out qPCR on a LightCycler^®^ 96 (Roche, Basel, Basel-Stadt, Switzerland) with the EVA Green I Kit in three technical replicates. We determined the qPCR efficiency by means of serial cDNA dilutions (standards). In line with the commonly accepted recommendations [76], we simultaneously analyzed four reference genes, namely: *B2m* (β-2-microglobulin) [241], *Hprt1* (hypoxanthine phosphoribosyltransferase 1) [242], *Ppia* (peptidylprolyl isomerase A) [243], and *Rpl30* (ribosomal protein L30) [244]. 

### 4.5. DEGs under Study 

In this work, we analyzed all the publicly available independent experimental RNA-Seq datasets—on transcriptomes from the tissues of hypertensive versus normotensive patients [26,27,28,29,30,31,32,33,34,35,36,37,38,39], hypertensive versus normotensive animals [7,40,41,42,43,44,45,46,47,48,49,50,51,52,53,54,55,56,57], and domestic versus wild animals [72,176,177,178,179,180,181,182,183,184,185,186,187,188,189,190,191,192,193].

### 4.6. Human Genes under Study

Here, we analyzed the 42 human genes that are orthologous to the 42 hippocampal DEGs of the tame versus aggressive rats (Table 2). Using the PubMed database [69], we characterized each of these 42 human genes in terms of what is already clinically known about how their underexpression or overexpression can manifest itself in hypertension (Table 9 and Tables S3 and S4). 

### 4.7. DNA Sequences under Study

For in silico analysis of the human genes encoding candidate molecular markers for hypertension that were for the first time suggested in this work, we retrieved both DNA sequences and SNPs of their 70 bp proximal promoters from the Ensembl database [209] and from the dbSNP database [210], respectively, relative to reference human genome assembly GRCh38/hg38 using the UCSC Genome Browser [206] in the dialog mode and additionally by means of toolbox BioPerl [207] in the automated mode, as shown in Figure S2.

### 4.8. In Silico Analysis of DNA Sequences

We examined SNPs within DNA sequences using our previously developed public web service SNP_TATA_Comparator [203], which applies our bioinformatic model of three-step binding between TBP and a human gene promoter, as detailed in the Supplementary Materials (i.e., Section S1 “Supplementary methods for DNA sequence analysis”) and additionally exemplified in Figure S2.

### 4.9. In Vitro Measurements

In this project, we in vitro measured K_D_ values expressed in “moles per liter” units of the equilibrium dissociation constant of TBP promoter complexes by means of the EMSA, for each of the nine chosen candidate SNP markers for hypertension subjected to this experimental verification—i.e., rs35518301:g, rs34166473:c, rs34500389:t, rs33980857:a, rs34598529:g, rs33931746:g, rs33931746:c, rs281864525:c, and rs63750953:deletion—as described in-depth in the Supplementary Materials (i.e., Section S2 “Supplementary methods for in vitro measurement”).

### 4.10. Knowledge Base on Domestic Animals’ DEGs with Orthologous Human Genes that Can Affect Hypertension

In files with the flat Excel-compatible textual format, here, on the one hand, we first documented all the suggested associations between DEGs (of domestic versus wild animals) homologous to the 42 DEGs (in the hippocampus of tame and aggressive rats) identified in this study. On the other hand, we documented how underexpression or overexpression of the human genes homologous to these hippocampal rat DEGs can affect hypertension. Next, using the MariaDB 10.2.12 web environment (MariaDB Corp AB, Espoo, Finland), we added the current findings to our previously created PetDEGsDB knowledge base, which is publicly available at www.sysbio.ru/domestic-wild (accessed on 16 January 2020).

### 4.11. Statistical Analysis

Using the options in the standard toolbox of Statistica (Statsoft^TM^), we applied the Mann–Whitney *U* test, Fisher’s Z-test, Pearson’s linear correlation test, the Goodman–Kruskal generalized correlation test, Spearman’s and Kendall’s rank correlation tests, Pearson’s χ^2^ test, Fisher’s exact test, and binomial-distribution analysis. 

Besides this, using the PAST4.04 software package [77], we conducted principal component analysis in the Bootstrap-refinement mode via its mode selection path “Multivariate” → “Ordination” → “Principal Components (PCA)” → “Correlation” → “Bootstrap.”

## 5. Conclusions

First of all, in this work, we performed high-throughput sequencing of the hippocampus transcriptome for three tame adult male rats compared with three aggressive ones (all unrelated animals). The primary experimental data are publicly available for those who would like to use them (NCBI SRA database ID: PRJNA668014) [75].

With the help of this transcriptome, we found the 42 hippocampal DEGs—in the tame versus aggressive rats in question—with statistical significance (P_ADJ_ < 0.05, Fisher’s Z-test with Benjamini’s correction for multiple comparisons) that was conventionally acceptable (Table 2). Moreover, we selectively validated these DEGs by independent experimental analyses (qPCR) of the other eight tame versus eight aggressive adult male rats from different unrelated litters of the same two outbred strains (Table 3 and Figure 1).

Besides this, using these 42 hippocampal tame-versus-aggressive rat DEGs, which reflect rat stress reactivity, we meta-analyzed (by homology) all the highly specific DEGs—of hypertensive versus normotensive subjects (i.e., patients and animals)—that we could find within mainstream hypertension-related transcriptomic research articles. First, we found significant correlations between stress reactivity-related and hypertension-related conventional log2 values (fold changes) of the homologous DEGs analyzed. Next, we found principal components, PC1 and PC2, corresponding to a half-difference and half-sum of these log2 values. Finally, these data pointed to downregulation of hemoglobin or β-protocadherins, corresponding to low blood viscosity [199] or a wide vascular inner diameter [200], as two hypertension theranostic molecular markers applicable to everyone, everywhere, anytime. 

## Figures and Tables

**Table 1:** Summary of searches for differentially expressed genes (DEGs) in hippocampal transcriptomes of three tame adult male rats (*Rattus norvegicus*) and three aggressive ones (all unrelated) in this work.

Group	Tame vs. Aggressive Rats
Total number of sequence reads (NCBI SRA ID: PRJNA668014)	169,529,658
Reads mapped to reference rat genome RGSC Rnor_6.0, UCSC Rn6, July 2014 (%)	146,521,467 (88.74%)
Expressed genes identified	14,039
Statistically significant DEGs (P_ADJ_ < 0.05, Fisher’s Z-test with Benjamini correction)	42

**Table 2:** The statistically significant DEGs in the hippocampus (of tame versus aggressive adult male rats) that were for the first time unidentified in this study.

#	Rat Gene, Name	*Symbol*	log2	*p*	*P_ADJ_*
1	Albumin	*Alb*	3.21	<10^−11^	<10^−7^
2	Aquaporin 1 (Colton blood group)	*Aqp1*	5.91	<10^−6^	<10^−2^
3	Achaete-scute family bHLH transcription factor 3	*Ascl3*	2.38	<10^−4^	<0.05
4	BAG cochaperone 3 (synonym: BCL2-associated athanogene 3)	*Bag3*	−0.92	<10^−4^	<0.05
5	BAR/IMD domain-containing adaptor protein 2-like 1	*Baiap2l1*	3.67	<10^−4^	<0.05
6	3-hydroxybutyrate dehydrogenase 1	*Bdh1*	0.40	<10^−4^	<0.05
7	Cholecystokinin B receptor	*Cckbr*	1.24	<10^−8^	<10^−4^
8	Chondroitin sulfate proteoglycan 4B	*Cspg4b*	3.47	<10^−4^	<0.05
9	Defensin β17	*Defb17*	5.94	<10^−4^	<0.05
10	Ectonucleotide pyrophosphatase/phosphodiesterase 2	*Enpp2*	2.41	<10^−3^	<0.05
11	Fras1-related extracellular matrix 1	*Frem1*	3.16	<10^−3^	<0.05
12	Glycerol-3-phosphate dehydrogenase 1	*Gpd1*	−1.34	<10^−6^	<10^−3^
13	Hemoglobin, β adult major chain	*Hbb-b1*	−6.19	<10^−7^	<10^−4^
14	Hepatocyte nuclear factor 4α	*Hnf4a*	6.51	<10^−3^	<0.05
15	5-hydroxytryptamine receptor 2C (synonym: serotonin receptor 2C)	*Htr2c*	2.03	<10^−3^	<0.05
16	Keratin 2	*Krt2*	−1.43	<10^−6^	<10^−3^
17	Leukocyte immunoglobulin-like receptor, subfamily B, member 3-like	*Lilrb3l*	7.45	<10^−4^	<0.05
18	Lymphocyte antigen 6 complex/Plaur domain-containing 1	*Lypd1*	−0.89	<10^−4^	<0.05
19	MORN repeat-containing 1	*Morn1*	1.42	<10^−11^	<10^−7^
20	Myomesin 2	*Myom2*	−1.24	<10^−4^	<0.05
21	Protocadherin β9	*Pcdhb9*	−1.03	<10^−4^	<0.05
22	Protocadherin γ subfamily A1	*Pcdhga1*	2.45	<10^−4^	<0.05
23	Prodynorphin	*Pdyn*	−0.89	<10^−4^	<0.05
24	Phospholipase A2, group IID	*Pla2g2d*	2.84	<10^−4^	<0.05
25	Phospholipase A2, group V	*Pla2g5*	3.85	<10^−4^	<0.05
26	Procollagen-lysine, 2-oxoglutarate 5-dioxygenase 1	*Plod1*	−0.67	<10^−3^	<0.05
27	Protein phosphatase 1, regulatory subunit 3B	*Ppp1r3b*	2.45	<10^−4^	<0.05
28	Prolactin receptor	*Prlr*	6.43	<10^−5^	<10^−2^
29	Glycogen phosphorylase L	*Pygl*	−1.21	<10^−5^	<0.05
30	RNA-binding motif protein 3	*Rbm3*	0.89	<10^−4^	<0.05
31	Retinol saturase	*Retsat*	−0.98	<10^−4^	<0.05
32	Solute carrier family 16, member 12	*Slc16a12*	3.08	<10^−3^	<0.05
33	Solute carrier family 4, member 5	*Slc4a5*	6.27	<10^−6^	<10^−3^
34	SPARC-related modular calcium-binding 2	*Smoc2*	−2.09	<10^−4^	<0.05
35	Serine peptidase inhibitor, Kunitz type 1	*Spint1*	−1.39	<10^−7^	<10^−4^
36	Sulfatase 1	*Sulf1*	3.72	<10^−6^	<10^−2^
37	Syncoilin, intermediate filament protein	*Sync*	1.17	<10^−3^	<0.05
38	Tandem C2 domains, nuclear	*Tc2n*	3.47	<10^−5^	<10^−2^
39	Tectorin α	*Tecta*	1.38	<10^−8^	<10^−5^
40	Transmembrane protein 60	*Tmem60*	0.79	<10^−4^	<0.05
41	Thioredoxin reductase 2	*Txnrd2*	−0.71	<10^−5^	<10^−2^
42	Uncoupling protein 2	*Ucp2*	0.73	<10^−4^	<0.05

Note. Hereinafter, log2: the log2-transformed fold change (i.e., ratio of an expression level of a given gene in tame rats to that in aggressive rats); *p* and *P_ADJ_*: statistical significance according to Fisher’s Z-test without and with the Benjamini correction for multiple comparisons, respectively.

**Table 3:** qPCR data on the selected DEGs from the hippocampus of the independently obtained eight tame adult male rats and eight other aggressive ones (all unrelated animals).

Design		Behavioral “Glove” Test [74] and the qPCR Data on Gene Expression [This Work]								
**Rat**	**Set**	**No. 1**	**2**	**3**	**4**	**5**	**6**	**7**	**8**	
**Glovetest**	**A**	−3	−3	−3	−3	−3	−3	−3	−3	
	**T**	3	3	3	3	3	3	3	3	
***DEG***	**Set**	**Relative expression with respect to four reference genes, qPCR, M_0_ ± SEM**								**TOTAL**
***Ascl3***	**A**	0.16 ± 0.02	0.88 ± 0.30	0.82 ± 0.08	0.09 ± 0.04	0.18 ± 0.03	0.07 ± 0.07	0.27 ± 0.11	0.32 ± 0.05	0.35 ± 0.17
	**T**	4.85 ± 4.38	3.40 ± 1.69	1.75 ± 0.24	2.21 ± 0.12	2.92 ± 0.05	4.48 ± 0.17	3.83 ± 0.33	2.64 ± 0.15	3.26 ± 1.71
***Defb17***	**A**	0.005 ± 0.005	0.01 ± 0.005	0.005 ± 0.005	0.005 ± 0.005	0.005 ± 0.005	ND	0.005 ± 0.005	0.005 ± 0.005	0.01 ± 0.01
	**T**	1.72 ± 0.04	3.22 ± 0.42	2.52 ± 0.14	1.82 ± 0.55	2.45 ± 0.10	4.43 ± 0.26	1.99 ± 0.89	2.34 ± 0.27	2.56 ± 0.53

Note. Sets: A, aggressive rats; T, tame rats; qPCR data: “M_0_ ± SEM” denotes the mean ± standard error of the mean for three technical replicates for each rat; ND, not detected.

**Table 4:** The DEGs—of hypertensive versus normotensive animals—that we could find (available in PubMed [69]).

#	Species	Hypertensive	Normotensive	Tissue	N_DEG_	Ref.
1	rat	OXYS	Wistar	hippocampus	85	[40]
2	rat	OXYS	Wistar	prefrontal cortex	73	[41]
3	rat	OXYS	Wistar	retina	85	[42]
4	rat	ISIAH	WAG	brain stem	206	[43]
5	rat	ISIAH	WAG	hypothalamus	137	[44]
6	rat	ISIAH	WAG	renal medulla	882	[45]
7	rat	ISIAH	WAG	renal cortex	309	[46]
8	rat	ISIAH	WAG	adrenal gland	1020	[47]
9	rat	SHR	Wistar	brain pericytes	21	[48]
10	rat	SHR	Wistar	kidney	35	[49]
11	rat	SD, monocrotaline-treated	SD, saline-treated	lung	10	[50]
12	rat	Dahl-SS, water after salt diet	Dahl-SS, QSYQ after salt diet	kidney	13	[51]
13	rat	*Resp18*-null Dahl-SS	Dahl-SS	kidney	14	[52]
14	rat	prenatal dexamethasone stress	norm	adrenal gland	93	[7]
15	mice	*Toxoplasma* infection in pregnancy	norm	uterus	10	[53]
16	mice	BPH/2J	BPN/3J	kidney	883	[54]
17	rabbit	G2K1C-treated	norm	middle cerebral artery	230	[55]
18	chicken	high (1.2%) Ca diet	normal (0.8%) Ca diet	kidney	92	[56]
19	chicken	cold stress with salt diet	healthy chicken	pulmonary arteries	18	[57]
*Σ*	4 species	14 animal models of human hypertension		14 tissues	4216	

Note. N_DEG_: the number of DEGs; BPH/2J, BPN/3J Dahl-SS, ISIAH, OXIS, SD, SHR, WAG, and Wistar: laboratory animal strains; QSYQ: QiShenYiQi pills, a cardioprotective remedy from traditional Chinese medicine; G2K1C: Goldblatt 2-kidney 1-clip; Ref.: reference.

**Table 5:** The analyzed DEGs—of hypertensive versus normotensive patients—that we could find (available in PubMed [69]).

*#*	Hypertensive	Normotensive	Tissue	N_DEG_	Ref.
1	renal medullary hypertension	norm	renal medulla	13	[26]
2	pulmonary arterial hypertension	norm	lung	49	[27]
3	pulmonary arterial hypertension	norm	lung	119	[28]
4	men with pulmonary arterial hypertension	normal men	blood	14	[29]
5	women with pulmonary arterial hypertension	normal women	blood	15	[29]
6	pulmonary hypertension during pulmonary fibrosis	norm	lung	3520	[30]
7	*BMPR2*-deficient human cells	normal cells	pulmonary artery endothelial cells	483	[31]
8	preeclampsia	normal pregnant	placenta	1228	[32]
9	preeclampsia	normal pregnant	placenta	10	[33]
10	preeclampsia	normal pregnant	venous blood	64	[34]
11	preeclampsia	normal pregnant	decidua basalis	372	[35]
12	excessive miR-210 in SWAN-71 cells	normal SWAN-71 cells	trophoblast cell line SWAN-71	19	[36]
13	hypertension-induced nephrosclerosis	norm	kidney	16	[37]
14	hypertension-related pre-invasive squamous cancer	normal cells, the same biopsies	squamous lung cancer cells	119	[38]
15	hypertension-induced atrial fibrillation	norm	auricle tissue biopsy	300	[39]
16	hypertension-induced coronary artery disease	norm	peripheral blood	1524	[39]
*Σ*	10 human hypertension-related disorders		12 tissues	7865	

Note. See the footnote of Table 4.

**Table 6:** Searching for hypertension-related molecular markers among the human genes orthologous to the 42 hippocampal DEGs (of tame versus aggressive rats) identified in this work. Here, we took into account the number of their homologous DEGs in the tissues of hypertensive versus normotensive subjects (patients and animals).

Rat Gene		Total Number of DEGs		Binomial Distribution		Rat Gene		Total Number of DEGs		Binomial Distribution	
*#*	*Symbol*	N_PC1_: Opposite Signs	N_PC2_: Matching Signs	*p*	*P*_ADJ_	*#*	*Symbol*	N_PC1_: Opposite Signs	N_PC2_: Matching Signs	*p*	*P*_ADJ_
i	ii	iii	iv	v	vi	i	ii	iii	iv	v	vi
*1*	*Alb*	1	1	0.75	1.00	*22*	*Pcdhga1*	1	1	0.75	1.00
*2*	*Aqp1*	6	6	0.61	1.00	*23*	*Pdyn*	0	0	ND	ND
*3*	*Ascl3*	1	1	0.75	1.00	*24*	*Pla2g2d*	19	12	0.14	1.00
*4*	*Bag3*	2	2	0.69	1.00	*25*	*Pla2g5*	19	12	0.14	1.00
*5*	*Baiap2l1*	1	0	0.50	1.00	*26*	*Plod1*	3	1	0.31	1.00
*6*	*Bdh1*	2	0	0.25	1.00	*27*	*Ppp1r3b*	2	3	0.50	1.00
*7*	*Cckbr*	1	0	0.50	1.00	*28*	*Prlr*	0	1	0.50	1.00
*8*	*Cspg4b*	0	0	ND	ND	*29*	*Pygl*	0	1	0.50	1.00
*9*	*Defb17*	2	3	0.50	1.00	*30*	*Rbm3*	15	12	0.35	1.00
*10*	*Enpp2*	3	8	0.11	1.00	*31*	*Retsat*	5	2	0.23	1.00
*11*	*Frem1*	1	1	0.75	1.00	*32*	*Slc16a12*	7	6	0.50	1.00
*12*	*Gpd1*	4	1	0.19	1.00	*33*	*Slc4a5*	7	5	0.83	1.00
*13*	*Hbb-b1*	24	3	10^−4^	10^−3^	*34*	*Smoc2*	3	1	0.31	1.00
*14*	*Hnf4a*	0	0	ND	ND	*35*	*Spint1*	1	1	0.75	1.00
*15*	*Htr2c*	3	3	0.65	1.00	*36*	*Sulf1*	0	0	ND	ND
*16*	*Krt2*	22	13	0.09	1.00	*37*	*Sync*	0	0	ND	ND
*17*	*Lilrb3l*	10	1	10^−2^	0.24	*38*	*Tc2n*	2	0	0.25	1.00
*18*	*Lypd1*	11	7	0.24	1.00	*39*	*Tecta*	0	1	0.50	1.00
*19*	*Morn1*	0	4	0.06	1.00	*40*	*Tmem60*	0	0	ND	ND
*20*	*Myom2*	2	1	0.50	1.00	*41*	*Txnrd2*	2	0	0.25	1.00
*21*	*Pcdhb9*	10	0	10^−3^	0.05	*42*	*Ucp2*	1	1	0.75	1.00

Note. *p* and ***P_AD_*_J_**: a significance estimate according to the binomial distribution without or with Bonferroni’s correction for multiple comparisons, respectively; ND: not detected; underlining: statistically significant hypertension-related molecular markers identified in this work.

**Table 7:** Statistically significant upregulation of the hemoglobin subunit and β-protocadherin DEGs—in the tissues of the hypertensive versus normotensive subjects (i.e., patients and animals)—that were for the first time compiled together here.

#	Species	Hypertensive	Normotensive	Tissue	*DEG*	log2	P_ADJ_	Ref.
i	ii	iii	iv	v	v	vi	vii	viii
*1*	rat	ISIAH	WAG	brain stem	*Hbb-b1*	1.42	10^−2^	[43]
*2*	rat	ISIAH	WAG	hypothalamus	*Hbb-b1*	2.02	10^−2^	[44]
*3*	rat	ISIAH	WAG	renal medulla	*Hbb-b1*	1.18	10^−2^	[45]
*4*	rat	ISIAH	WAG	adrenal gland	*Hbb-b1*	1.32	10^−2^	[47]
*5*	rat	ISIAH	WAG	adrenal gland	*Hba2*	0.69	10^−2^	[47]
*6*	rat	ISIAH	WAG	adrenal gland	*Hbb*	2.02	10^−2^	[47]
*7*	rat	ISIAH	WAG	adrenal gland	*Hbb-m*	3.78	10^−2^	[47]
*8*	rat	ISIAH	WAG	brain stem	*Hba2*	0.58	0.05	[43]
*9*	rat	ISIAH	WAG	brain stem	*Hbb*	1.88	10^−2^	[43]
*10*	rat	ISIAH	WAG	brain stem	*Hbb-m*	3.65	10^−2^	[43]
*11*	rat	ISIAH	WAG	hypothalamus	*Hba1*	1.14	10^−2^	[44]
*12*	rat	ISIAH	WAG	hypothalamus	*Hba2*	1.32	10^−2^	[44]
*13*	rat	ISIAH	WAG	hypothalamus	*Hbb*	3.23	10^−2^	[44]
*14*	rat	ISIAH	WAG	hypothalamus	*Hbb-m*	1.09	10^−2^	[44]
*15*	rat	ISIAH	WAG	renal medulla	*Hbb*	−0.68	10^−2^	[45]
*16*	rat	ISIAH	WAG	renal medulla	*Hbb-m*	2.72	10^−2^	[45]
*17*	rat	ISIAH	WAG	renal medulla	*Hbb-s*	2.38	10^−2^	[45]
*18*	human	preeclampsia	norm	placenta	*HBD*	−0.63	10^−3^	[32]
*19*	human	pulmonary hypertension during pulmonary fibrosis	norm	lungs	*HBD*	−2.83	10^−3^	[30]
*20*	human	pulmonary hypertension	norm	lungs	*HBA1*	2.08	10^−9^	[28]
*21*	human	pulmonary hypertension	norm	lungs	*HBB*	2.46	10^−10^	[28]
*22*	human	HT-induced coronary disease	norm	peripheral blood	*HBBP1*	1.03	0.05	[39]
*23*	human	HT-induced coronary disease	norm	peripheral blood	*HBE1*	1.42	0.05	[39]
*24*	human	HT-induced coronary disease	norm	peripheral blood	*HBG2*	4.49	0.05	[39]
*25*	human	HT-induced coronary disease	norm	peripheral blood	*HBM*	5.33	0.05	[39]
*26*	human	HT-induced coronary disease	norm	peripheral blood	*HBQ1*	3.10	0.05	[39]
*27*	human	HT-induced atrial fibrillation	norm	auricle tissue biopsy	*HBA2*	2.37	10^−2^	[39]
*28*	rat	ISIAH	WAG	brain stem	*Pcdhb7*	1.60	10^−2^	[43]
*29*	mouse	BPH/2J	BPN/3J	kidneys	*Pcdhb16*	1.22	10^−3^	[54]
*30*	human	pulmonary hypertension during pulmonary fibrosis	norm	lungs	*PCDHB10*	1.89	10^−2^	[30]
*31*	human	pulmonary hypertension during pulmonary fibrosis	norm	lungs	*PCDHB15*	1.47	10^−4^	[30]
*32*	human	pulmonary hypertension during pulmonary fibrosis	norm	lungs	*PCDHB16*	1.38	10^−4^	[30]
*33*	human	pulmonary hypertension during pulmonary fibrosis	norm	lungs	*PCDHB17P*	1.21	10^−2^	[30]
*34*	human	pulmonary hypertension during pulmonary fibrosis	norm	lungs	*PCDHB4*	2.93	10^−4^	[30]
*35*	human	pulmonary hypertension during pulmonary fibrosis	norm	lungs	*PCDHB6*	1.35	10^−2^	[30]
*36*	human	HT-induced coronary disease	norm	peripheral blood	*PCDHB11*	1.12	0.05	[39]
*37*	human	HT-induced coronary disease	norm	peripheral blood	*PCDHB13*	1.04	0.05	[39]

Notes. HT, hypertension.

**Table 8:** The investigated genome-wide RNA-Seq transcriptomes (of domestic animals with their wild congeners) that we could find in the PubMed database [69].

*#*	Domestic Animals	Wild Animals	Tissue	N_DEG_	Ref.
1	tame rats	aggressive rats	hypothalamus	46	[72]
2	tame rats	aggressive rats	frontal cortex	20	[187]
3	guinea pigs	cavy	frontal cortex	883	[187]
4	domestic rabbits	wild rabbits	frontal cortex	17	[187]
5	domestic rabbits	wild rabbits	parietal-temporal cortex	216	[188]
6	domestic rabbits	wild rabbits	amygdala	118	[188]
7	domestic rabbits	wild rabbits	hypothalamus	43	[188]
8	domestic rabbits	wild rabbits	hippocampus	100	[188]
9	dogs	wolves	blood	450	[189]
10	dogs	wolves	frontal cortex	13	[187]
11	tame foxes	aggressive foxes	pituitary	327	[190]
12	pigs	boars	frontal cortex	30	[187]
13	pigs	boars	frontal cortex	34	[191]
14	pigs	boars	pituitary	22	[192]
15	domestic chicken	wild chicken	pituitary	474	[193]
*Σ*	7 domestic animal species	7 wild animal species	8 tissues	2393	

**Table 9:** Comparing the effects of changes to the expression of homologous genes (a) on hypertension development in humans and (b) during the divergence of domestic and wild animals from their most recent common ancestors.

(a) Humans					(b) Animals					
*Gene*	Effect of Gene Expression Changes on Hypertension (HT): Hypertensive (→) or Normotensive (←)				RNA-Seq		Effect of Gene Expression Changes during Divergence from the Most Recent Common Ancestor			Ref.
	Downregulation	HT	Upregulation	HT	*DEG*	log2	Downregulation	Upregulation	Tissue	
i	ii	iii	iv	v	vi	vii	viii	ix	x	xi
*HBB, HBD*	low blood viscosity [199]	←	high-altitude environment provokes hyperhemoglobinemia and hypertension [103]	→	*Hbb-b1*	−6.19	tame rat	aggressive rat	hippocampus	[this work]
					*Hbb-b1*	−3.97	tame rat	aggressive rat	hypothalamus	[72]
					*Hbbl*	−5.92	dogs	wolves	blood	[189]
					*Hba1*	−4.06	dogs	wolves	blood	[189]
					*Hbad*	−1.07	domestic chickens	wild chickens	pituitary	[193]
					*Hbm*	−6.46	dogs	wolves	blood	[189]
					*Hbz1*	−7.10	dogs	wolves	blood	[189]
*PCDHB9*	wide vascular inner diameter [200]	←	higher risks of gastric cancer [93], surgical removal of which relieves hypertension [12]	→	*Pcdhb9*	−1.03	tame rat	aggressive rat	hippocampus	[this work]
					*Pcdhb9*	−1.01	tame rat	aggressive rat	hypothalamus	[72]
					*Pcdhb15*	−1.04	domestic rabbits	wild rabbits	parietal-temporal cortex	[188]

**Table 10:** Correlations between the effects of unidirectional changes in the expression of homologous genes (a) on human hypertension and (b) during the divergence of studied domestic and wild animals from their most recent common ancestor.

	(a) Humans	Effect of Expression Changes of Genes Encoding Hemoglobin Subunits and β-Protocadherins in Patients		Binomial Distribution	Pearson’s χ^2^ Test		Fisher’s Exact Test
(b) Animals		Hypertensive	Normotensive		χ^2^	*p*	
Effect of expression changes of genes encoding hemoglobin subunits and β-protocadherins during animal microevolution	wild	10	0	10^−4^	20.00	10^−3^	10^−5^
	domestic	0	10	10^−4^			

**Table 11:** The hypertension-related candidate SNP markers within *HBB*, *HBD,* and *PCDHB9* promoters (predicted here) and their comparison with genome-wide patterns.

Data: GRCh38, dbSNP rel. 153 [210]				H_0_: Neutral Drift [229,230]			H_0_: “→HT and ←HT Equivalence”		
SNPs	N_GENE_	N_SNP_	N_RES_	N_>_	N_<_	*p*(H_0_: N_>_ < N_<_) [227]	N_→HT_	N_←HT_	*p*(H_0_: N_→HT_ ≡ N_←HT_ )
Whole-genome norm for SNPs of TBP sites [228]	10^4^	10^5^	10^3^	200	800	>0.99	-	-	-
HT-related candidate SNP markers at TBP sites [this work]	3	85	27	8	19	>0.99	8	19	<0.05

Notes. Hypertension (HT): normotensive (←HT) and hypertensive (→HT). N_GENE_ and N_SNP_: total numbers of the human genes and of their SNPs meeting the criteria for this study. N_RES_: the total number of the candidate SNP markers that can increase (N_>_) or decrease (N_<_) the affinity of TATA-binding protein (TBP) for these promoters and to respectively affect the expression of these genes. N_←HT_ and N_→HT_: total numbers of the candidate SNP markers that can prevent or provoke hypertension. *p*(H_0_): the estimate of probability for the acceptance of this H_0_ hypothesis, in accordance with the binomial distribution. TBP-site: TATA-binding-protein binding site.

**Table 12:** qPCR primers selected using publicly available Web service PrimerBLAST [240].

No.	*Gene*	Forward, 5′→3′	Reverse, 5′→3′
**DEGs identified in hippocampus of tame versus aggressive adult male rats [this work]**			
1	*Ascl3*	CCTCTGCTGCCCTTTTCCAG	ACTTGACTCGCTGCCTCTCT
2	*Defb17*	TGGTAGCTTGGACTTGAGGAAAGAA	TGCAGCAGTGTGTTCCAGGTC
**Reference genes**			
3	*B2m*	GTGTCTCAGTTCCACCCACC	TTACATGTCTCGGTCCCAGG
4	*Hprt1*	TCCCAGCGTCGTGATTAGTGA	CCTTCATGACATCTCGAGCAAG
5	*Ppia*	TTCCAGGATTCATGTGCCAG	CTTGCCATCCAGCCACTC
6	*Rpl30*	CATCTTGGCGTCTGATCTTG	TCAGAGTCTGTTTGTACCCC

Notes. Regarding the DEGs subjected to this qPCR verification, see Table 2; reference rat genes: *B2m*, β-2-microglobulin [241]; *Hprt1*, hypoxanthine phosphoribosyltransferase 1 [242]; *Ppia*, peptidylprolyl isomerase A [243]; *Rpl30*, ribosomal protein L30 [244].

## Data Availability

The primary RNA-Seq data obtained in this work were deposited in the NCBI SRA database (ID = PRJNA668014).

## Supplementary Materials

Supplementary materials can be found at https://www.mdpi.com/article/10.3390/ijms23052835/s1.

## Abbreviations

DEG	differentially expressed gene
EMSA	electrophoretic mobility shift assay
HT	hypertension
log2 value	log_2_-transformed gene expression fold change
PC1 (PC2)	major (minor) principal component
qPCR	quantitative polymerase chain reaction
RNA-Seq	RNA sequencing
SNP	single-nucleotide polymorphism
TBP	TATA-binding protein
