# Supplementary material for: Stress Reactivity, Susceptibility to Hypertension, and Differential Expression of Genes in Hypertensive Compared to Normotensive Patients
Source: Int J Mol Sci. 2022 Mar 4;23(5):2835. doi: 10.3390/ijms23052835 (PMC8911431; doi:10.3390/ijms23052835)
Supplement: Supplementary file 1 [file ijms-23-02835-s001.zip › ijms-1557167-supplementary.pdf]

International Journal of
Molecular Sciences

MDPI

Supplementary Materials

Stress Reactivity, Susceptibility to Hypertension, and Differential Expression of Genes in Hypertensive Compared to Normotensive Patients

Dmitry Oshchepkov[1], Irina Chadaeva[1], Rimma Kozhemyakina[1], Karina Zolotareva[1], Bato Khandaev[1], Ekaterina Sharypova[1], Petr Ponomarenko[1], Anton Bogomolov[1], Natalya Klimova[1], Svetlana Shikhevich[1], Olga Redina[1], Nataliya G. Kolosova[1], Maria Nazarenko[2], Arcady Markel[1], Mikhail Ponomarenko[1,*]

[1] Institute of Cytology and Genetics, Novosibirsk 630090, Russia
[2] Institute of Medical Genetics, Tomsk National Research Medical Center, Tomsk, 634009, Russia;
[*] Correspondence: pon@bionet.nsc.ru. Tel.: +7 (383) 363-49-63 ext. 1311 (M.P.)

Abstract: Although half of hypertensive patients have hypertensive parents, known hypertension-related human loci identified by genome-wide analysis explain only 3% of hypertension heredity. Therefore, mainstream transcriptome profiling of hypertensive subjects addresses differentially expressed genes (DEGs) specific to gender, age, and comorbidities in accordance with predictive preventive personalized participatory medicine treating patients according to symptoms, an individual lifestyle, and genetic background. Within this mainstream paradigm, here we determined whether among known hypertension-related DEGs that we could find, there is any genome-wide hypertension theranostic molecular marker applicable to everyone everywhere anytime. Therefore, we sequenced the hippocampal transcriptome of tame and aggressive rats, corresponding to low and high stress reactivity, whose increase raises hypertension risk; we identified stress-reactivity-related rat DEGs and compared them with their known homologous hypertension-related animal DEGs. This yielded significant correlations between stress-reactivity-related and hypertension-related fold changes (log_2-values) of these DEG homologs. We found principal components, PC1 and PC2, corresponding to a half-difference and half-sum of these log_2-values. Using the DEGs of hypertensive versus normotensive patients (as control), we verified the correlations and principal components. This analysis highlighted downregulation of β-protocadherins and hemoglobin as whole-genome hypertension theranostic molecular markers associated with a wide vascular inner diameter and low blood viscosity, respectively.

CONTENT

Figure S1. The comparison of the known DEGs (of hypertensive versus normotensive animals, as reported by others) that we could find with their homologous DEGs (in the hippocampus of tame versus aggressive rats) found here.

Table S1. The hippocampal DEGs—of tame versus aggressive rats—found in this work in comparison with their known homologous DEGs (in hypertensive versus normotensive animals as reported by others) that we could find.

Table S2. The tame-versus-aggressive-rat hippocampal DEGs found in this work in comparison with their known homologous DEGs—in hypertensive versus normotensive patients as reported by others—that we could find.

Table S3. Effects—on hypertension—of underexpression or overexpression of the human genes homologous to the DEGs (in the hippocampus of tame versus aggressive rats) identified in this work according to the current state of the PubMed database [69].

Section S1. Supplementary methods for DNA sequence analysis

Figure S2. An illustrative example of the result produced by SNP_TATA_Comparator [203] in the case of the hypertension-related candidate SNP marker (rs34166473) reducing blood viscosity via *HBD* downregulation.

Table S4. Hypertension-related candidate SNP markers within the 70 bp proximal promoter of human genes *HBB*, *HBD*, and *PCDHB9* as calculated here.

Section S2. Supplementary methods for *in vitro* measurements

Table S5. ODNs identical to fragments of ancestral (WT) and minor (SNP ID) variants of the human gene promoters studied in this work

Figure S3. Statistically significant correlations between *in silico*–predicted and *in vitro*–measured values of equilibrium dissociation constant K_D of TBP–promoter affinity expressed in "moles per liter" units converted to a natural logarithm scale, ln units.

References. Hereinafter see section "References" of the main text.

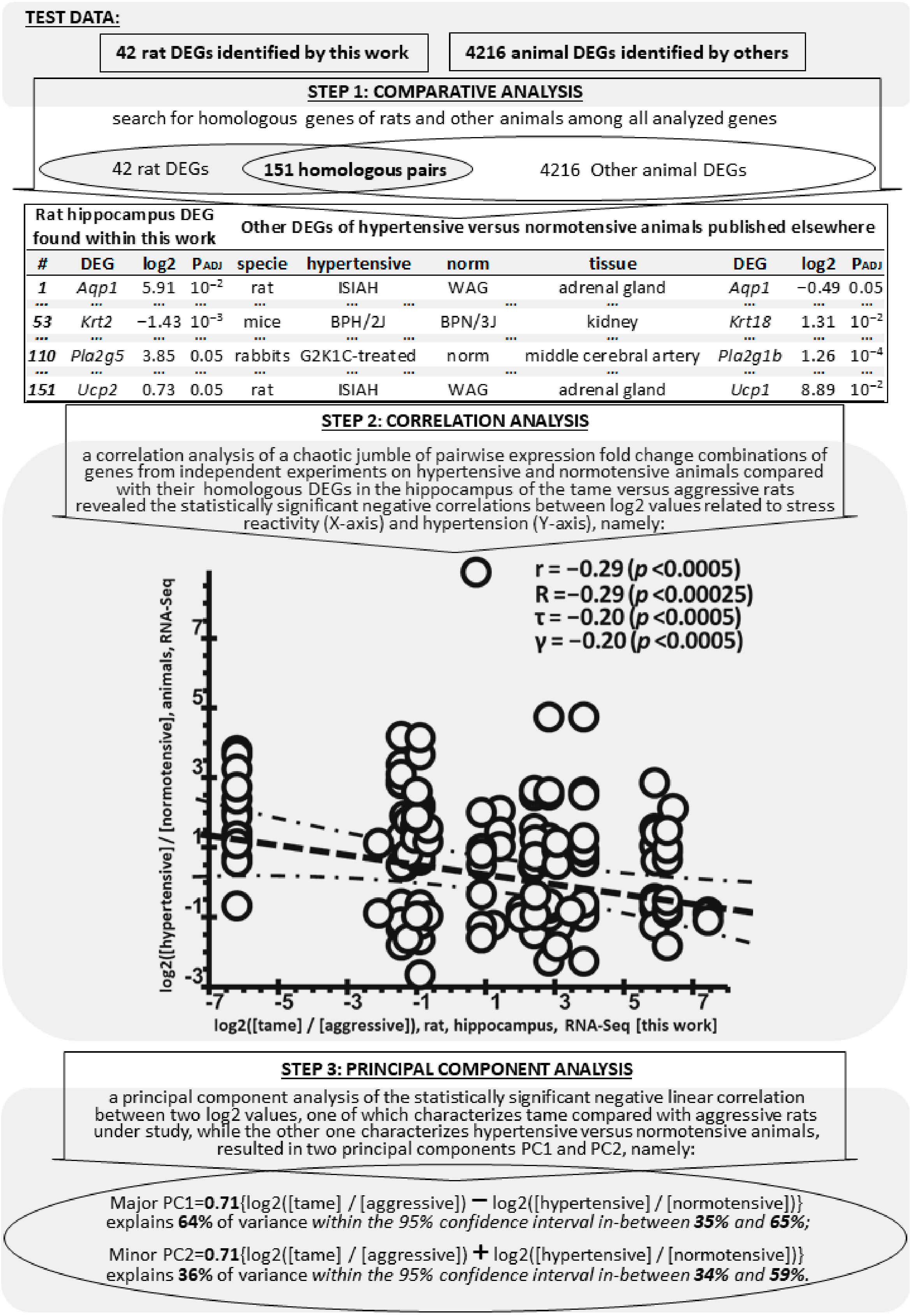

Figure S1. The comparison of the known DEGs (of hypertensive versus normotensive animals, as reported by others) that we could find with their homologous DEGs (in the hippocampus of tame versus aggressive rats) found here. *Legend*: see the footnote of Table S1; the X- and Y-axes correspond to columns iii and ix of Table S1; circles correspond to rows of Table S1; Dashed and dash-and-dot lines denote linear regression and boundaries of its 95% confidence interval calculated in the Statistica software (Statsoft™, Tulsa, OK, USA). r, R, τ, γ, and *p* are coefficients of Pearson's linear correlation, Spearman's rank correlation, Kendall's rank correlation, Goodman–Kruskal generalized correlation, and their p values (statistical significance), respectively, calculated by means of the Statistica software (Statsoft™); PC1 and PC2: principal components calculated in the Bootstrap-based refinement mode using the PAST4.04 software [77].

Table S1. The hippocampal DEGs—of tame versus aggressive rats—found in this work in comparison with their known homologous DEGs (in hypertensive versus normotensive animals as reported by others) that we could find.

#	hippocampus, tame *vs* aggressive rats			DEGs in the tissues of the hypertensive *vs* normotensive animals as said by others that we could find							
	DEG	**log2₂**	P_{ADJ}	species	**hypertensive**	**normotensive**	**tissue**	***DEG***	**log2₂**	P_{ADJ}	Ref
**i**	**ii**	**iii**	**iv**	**v**	**vi**	**vii**	**viii**	**ix**	**x**	**xi**	xii
1	*Aqp1*	5.91	10^{-2}	rat	ISIAH	WAG	adrenal gland	*Aqp1*	−0.49	0.05	[47]
2	*Aqp1*	5.91	10^{-2}	rat	SHR	Wistar	brain pericytes	*Aqp1*	2.89	0.05	[48]
3	*Aqp1*	5.91	10^{-2}	rat	ISIAH	WAG	adrenal gland	*Aqp7*	1.04	0.05	[47]
4	*Aqp1*	5.91	10^{-2}	rat	ISIAH	WAG	renal medulla	*Aqp2*	−0.47	0.05	[45]
5	*Aqp1*	5.91	10^{-2}	rat	ISIAH	WAG	renal medulla	*Aqp3*	−0.77	10^{-2}	[45]
6	*Aqp1*	5.91	10^{-2}	rat	ISIAH	WAG	renal medulla	*Aqp5*	−0.59	0.05	[45]
7	*Aqp1*	5.91	10^{-2}	mice	BPH/2J	BPN/3J	kidney	*Aqp6*	1.53	0.05	[54]
8	*Aqp1*	5.91	10^{-2}	mice	BPH/2J	BPN/3J	kidney	*Aqp7*	−1.26	10^{-3}	[54]
9	*Ascl3*	2.38	0.05	rat	OXYS	Wistar	prefrontal cortex	*Ascl1*	0.44	10^{-2}	[41]
10	*Cckbr*	1.24	10^{-4}	rat	ISIAH	WAG	brain stem	*Cckbr*	−1.25	10^{-2}	[43]
11	*Defb17*	5.94	0.05	rat	ISIAH	WAG	renal cortex	*Defb1*	0.59	0.05	[46]
12	*Defb17*	5.94	0.05	chicken	high Ca-diet	normal Ca-diet	kidney	*Defb4a*	1.41	10^{-2}	[56]
13	*Enpp2*	2.41	0.05	rat	ISIAH	WAG	renal medulla	*Enpp2*	0.68	10^{-2}	[45]
14	*Enpp2*	2.41	0.05	rat	ISIAH	WAG	renal medulla	*Enpp3*	0.71	10^{-2}	[45]
15	*Enpp2*	2.41	0.05	rat	ISIAH	WAG	adrenal gland	*Enpp3*	0.50	0.05	[47]
16	*Enpp2*	2.41	0.05	rat	ISIAH	WAG	renal medulla	*Enpp6*	2.47	10^{-2}	[45]
17	*Enpp2*	2.41	0.05	rat	ISIAH	WAG	brain stem	*Enpp6*	1.53	10^{-2}	[43]
18	*Enpp2*	2.41	0.05	rat	ISIAH	WAG	renal cortex	*Enpp6*	2.62	0.05	[46]
19	*Enpp2*	2.41	0.05	mice	BPH/2J	BPN/3J	kidney	*Enpp6*	−1.48	10^{-4}	[54]
20	*Frem1*	3.16	0.05	mice	BPH/2J	BPN/3J	kidney	*Frem1*	−1.29	0.05	[54]
21	*Gpd1*	−1.34	10^{-3}	rat	ISIAH	WAG	renal medulla	*Gpd1*	0.44	0.05	[45]
22	*Gpd1*	−1.34	10^{-3}	rat	Prenatal dexamethasone-stressed	norm	adrenal gland	*Gpd1l*	1.90	0.05	[7]
23	*Hbb-b1*	−6.19	10^{-4}	rat	ISIAH	WAG	brain stem	*Hbb-b1*	1.42	10^{-2}	[43]
24	*Hbb-b1*	−6.19	10^{-4}	rat	ISIAH	WAG	hypothalamus	*Hbb-b1*	2.02	10^{-2}	[44]
25	*Hbb-b1*	−6.19	10^{-4}	rat	ISIAH	WAG	renal medulla	*Hbb-b1*	1.18	10^{-2}	[45]
26	*Hbb-b1*	−6.19	10^{-4}	rat	ISIAH	WAG	adrenal gland	*Hbb-b1*	1.32	10^{-2}	[47]
27	*Hbb-b1*	−6.19	10^{-4}	rat	ISIAH	WAG	adrenal gland	*Hba2*	0.69	10^{-2}	[47]
28	*Hbb-b1*	−6.19	10^{-4}	rat	ISIAH	WAG	adrenal gland	*Hbb*	2.02	10^{-2}	[47]
29	*Hbb-b1*	−6.19	10^{-4}	rat	ISIAH	WAG	adrenal gland	*Hbb-m*	3.78	10^{-2}	[47]
30	*Hbb-b1*	−6.19	10^{-4}	rat	ISIAH	WAG	brain stem	*Hba2*	0.58	0.05	[43]
31	*Hbb-b1*	−6.19	10^{-4}	rat	ISIAH	WAG	brain stem	*Hbb*	1.88	10^{-2}	[43]

Table S1. *Cont.*

#	hippocampus, tame *vs* aggressive rats			DEGs in the tissues of the hypertensive *vs* normotensive animals as said by others that we could find							
	DEG	**log2₂**	P_{ADJ}	species	**hypertensive**	**normotensive**	**tissue**	***DEG***	**log2₂**	P_{ADJ}	Ref
**i**	**ii**	**iii**	**iv**	**v**	**vi**	**vii**	**viii**	**ix**	**x**	**xi**	xii
32	*Hbb-b1*	−6.19	10^{-4}	rat	ISIAH	WAG	brain stem	*Hbb-m*	3.65	10^{-2}	[43]
33	*Hbb-b1*	−6.19	10^{-4}	rat	ISIAH	WAG	hypothalamus	*Hba1*	1.14	10^{-2}	[44]
34	*Hbb-b1*	−6.19	10^{-4}	rat	ISIAH	WAG	hypothalamus	*Hba2*	1.32	10^{-2}	[44]
35	*Hbb-b1*	−6.19	10^{-4}	rat	ISIAH	WAG	hypothalamus	*Hbb*	3.23	10^{-2}	[44]
36	*Hbb-b1*	−6.19	10^{-4}	rat	ISIAH	WAG	hypothalamus	*Hbb-m*	1.09	10^{-2}	[44]
37	*Hbb-b1*	−6.19	10^{-4}	rat	ISIAH	WAG	renal medulla	*Hbb*	−0.68	10^{-2}	[45]
38	*Hbb-b1*	−6.19	10^{-4}	rat	ISIAH	WAG	renal medulla	*Hbb-m*	2.72	10^{-2}	[45]
39	*Hbb-b1*	−6.19	10^{-4}	rat	ISIAH	WAG	renal medulla	*Hbb-s*	2.38	10^{-2}	[45]
40	*Htr2c*	2.03	0.05	rat	OXIS	Wistar	hippocampus	*Htr2c*	−0.80	10^{-3}	[40]
41	*Htr2c*	2.03	0.05	rabbit	Goldblatt 2-kidney 1-clip exposed	norm	middle cerebral artery	*Htr4*	−1.01	10^{-4}	[55]
42	*Krt2*	−1.43	10^{-3}	rat	ISIAH	WAG	renal medulla	*Krt15*	2.90	0.05	[45]
43	*Krt2*	−1.43	10^{-3}	rat	ISIAH	WAG	renal medulla	*Krt19*	4.18	10^{-2}	[45]
44	*Krt2*	−1.43	10^{-3}	rat	ISIAH	WAG	renal medulla	*Krt23*	−1.33	0.05	[45]
45	*Krt2*	−1.43	10^{-3}	rat	ISIAH	WAG	renal medulla	*Krt8*	0.49	0.05	[45]
46	*Krt2*	−1.43	10^{-3}	rat	ISIAH	WAG	renal cortex	*Krt19*	3.37	0.05	[46]
47	*Krt2*	−1.43	10^{-3}	rat	ISIAH	WAG	renal cortex	*Krt8*	0.47	0.05	[46]
48	*Krt2*	−1.43	10^{-3}	rat	ISIAH	WAG	adrenal gland	*Krt10*	−1.82	10^{-2}	[47]
49	*Krt2*	−1.43	10^{-3}	rat	ISIAH	WAG	adrenal gland	*Krt18*	2.82	10^{-2}	[47]
50	*Krt2*	−1.43	10^{-3}	rat	ISIAH	WAG	adrenal gland	*Krt8*	−0.63	0.05	[47]
51	*Krt2*	−1.43	10^{-3}	rat	SD, monocrotaline treated	SD, norm	lung	*Krt12*	2.87	10^{-2}	[50]
52	*Krt2*	−1.43	10^{-3}	mice	prepregnancy toxoplasma-treated	norm	uterus	*Krt8*	3.09	0.05	[53]
53	*Krt2*	−1.43	10^{-3}	mice	BPH/2J	BPN/3J	kidney	*Krt18*	1.31	10^{-2}	[54]
54	*Krt2*	−1.43	10^{-3}	rabbit	Goldblatt 2-kidney 1-clip exposed	norm	middle cerebral artery	*Krt7*	1.21	10^{-4}	[55]
55	*Krt2*	−1.43	10^{-3}	rabbit	Goldblatt 2-kidney 1-clip exposed	norm	middle cerebral artery	*Krt84*	−1.15	10^{-4}	[55]
56	*Lilrb3l*	7.45	0.05	rat	ISIAH	WAG	adrenal gland	*Lilrb3l*	−0.85	10^{-2}	[47]
57	*Lilrb3l*	7.45	0.05	rat	ISIAH	WAG	renal medulla	*Lilrb4*	−0.93	0.05	[45]
58	*Lilrb3l*	7.45	0.05	rat	ISIAH	WAG	renal medulla	*Lilra5*	−1.11	10^{-2}	[45]
59	*Lypd1*	−0.89	0.05	rat	ISIAH	WAG	renal medulla	*Ly6al*	3.65	10^{-2}	[45]
60	*Lypd1*	−0.89	0.05	rat	ISIAH	WAG	renal medulla	*Ly6i*	−1.66	10^{-2}	[45]
61	*Lypd1*	−0.89	0.05	rat	ISIAH	WAG	renal medulla	*Ly6l*	2.13	10^{-2}	[45]
62	*Lypd1*	−0.89	0.05	rat	ISIAH	WAG	renal medulla	*Ly6-l*	0.82	10^{-2}	[45]
63	*Lypd1*	−0.89	0.05	rat	ISIAH	WAG	renal medulla	*Lynx1*	−0.65	10^{-2}	[45]

Table S1. *Cont.*

#	hippocampus, tame *vs* aggressive rats			DEGs in the tissues of the hypertensive *vs* normotensive animals as said by others that we could find							
	DEG	**log2**$_2$	P_{ADJ}	species	**hypertensive**	**normotensive**	**tissue**	***DEG***	**log2**$_2$	P_{ADJ}	Ref
**i**	**ii**	**iii**	**iv**	**v**	**vi**	**vii**	**viii**	**ix**	**x**	**xi**	xii
64	*Lypd1*	−0.89	0.05	rat	ISIAH	WAG	renal cortex	*Ly6al*	4.14	0.05	[46]
65	*Lypd1*	−0.89	0.05	rat	ISIAH	WAG	adrenal gland	*Ly6bl*	0.63	0.05	[47]
66	*Lypd1*	−0.89	0.05	rat	ISIAH	WAG	adrenal gland	*Ly6e*	2.21	10^{-2}	[47]
67	*Lypd1*	−0.89	0.05	rat	ISIAH	WAG	adrenal gland	*Lynx1*	0.70	10^{-2}	[47]
68	*Lypd1*	−0.89	0.05	rat	OXIS	Wistar	retina	*Ly6c*	−2.65	0.05	[42]
69	*Lypd1*	−0.89	0.05	rat	OXIS	Wistar	retina	*Ly6g6e*	−1.17	10^{-4}	[42]
70	*Lypd1*	−0.89	0.05	mice	BPH/2J	BPN/3J	kidney	*Ly6f*	−1.22	0.05	[54]
71	*Lypd1*	−0.89	0.05	rabbit	Goldblatt 2-kidney 1-clip exposed	norm	middle cerebral artery	*Ly6g5c*	1.32	10^{-4}	[55]
72	*Morn1*	1.42	10^{-3}	rat	ISIAH	WAG	brain stem	*Morn1*	2.05	10^{-2}	[43]
73	*Morn1*	1.42	10^{-3}	rat	ISIAH	WAG	hypothalamus	*Morn1*	1.44	10^{-2}	[44]
74	*Morn1*	1.42	10^{-3}	rat	ISIAH	WAG	adrenal gland	*Morn1*	1.01	10^{-2}	[47]
75	*Myom2*	−1.24	0.05	rat	ISIAH	WAG	adrenal gland	*Myom2*	0.81	10^{-2}	[47]
76	*Myom2*	−1.24	0.05	rat	Prenatal dexamethasone-stressed	norm	adrenal gland	*Myom2*	1.75	0.05	[7]
77	*Myom2*	−1.24	0.05	rat	ISIAH	WAG	brain stem	*Myom3*	−1.62	10^{-2}	[43]
78	*Pcdhb9*	−1.03	0.05	rat	ISIAH	WAG	brain stem	*Pcdhb7*	1.60	10^{-2}	[43]
79	*Pcdhb9*	−1.03	0.05	mice	BPH/2J	BPN/3J	kidney	*Pcdhb16*	1.22	10^{-3}	[54]
80	*Pla2g2d*	2.84	0.05	rat	ISIAH	WAG	adrenal gland	*Pla2g2d*	2.49	10^{-2}	[47]
81	*Pla2g2d*	2.84	0.05	rat	SHR	Wistar	brain pericytes	*Pla2g2a*	4.74	0.05	[48]
82	*Pla2g2d*	2.84	0.05	rat	ISIAH	WAG	adrenal gland	*Pla2g16*	0.62	10^{-2}	[47]
83	*Pla2g2d*	2.84	0.05	rat	ISIAH	WAG	adrenal gland	*Pla2g7*	−0.97	10^{-2}	[47]
84	*Pla2g2d*	2.84	0.05	rat	ISIAH	WAG	brain stem	*Pla2g3*	−0.84	10^{-2}	[43]
85	*Pla2g2d*	2.84	0.05	rat	ISIAH	WAG	brain stem	*Pld5*	−0.99	10^{-2}	[43]
86	*Pla2g2d*	2.84	0.05	rat	ISIAH	WAG	hypothalamus	*Pld5*	−0.77	10^{-2}	[44]
87	*Pla2g2d*	2.84	0.05	rat	ISIAH	WAG	renal medulla	*Pla2g4a*	−0.69	10^{-2}	[45]
88	*Pla2g2d*	2.84	0.05	rat	ISIAH	WAG	renal medulla	*Pld6*	0.87	0.05	[45]
89	*Pla2g2d*	2.84	0.05	rat	ISIAH	WAG	renal cortex	*Pla2g16*	0.58	0.05	[46]
90	*Pla2g2d*	2.84	0.05	rat	ISIAH	WAG	renal cortex	*Pla2g7*	0.71	0.05	[46]
91	*Pla2g2d*	2.84	0.05	rat	OXIS	Wistar	retina	*Plaat1*	−2.28	10^{-7}	[42]
92	*Pla2g2d*	2.84	0.05	rat	OXIS	Wistar	hippocampus	*Pla2g6*	0.93	10^{-5}	[40]
93	*Pla2g2d*	2.84	0.05	rat	OXIS	Wistar	hippocampus	*Plcg2*	−0.61	10^{-2}	[40]
94	*Pla2g2d*	2.84	0.05	mice	BPH/2J	BPN/3J	kidney	*Pla2g5*	2.59	10^{-6}	[54]
95	*Pla2g2d*	2.84	0.05	rabbit	Goldblatt 2-kidney 1-clip exposed	norm	middle cerebral artery	*Pla2g1b*	1.26	10^{-4}	[55]

Table S1. *Cont.*

#	hippocampus, tame vs aggressive rats			DEGs in the tissues of the hypertensive vs normotensive animals as said by others that we could find							
	**DEG**	**log2₂**	P_{ADJ}	species	**hypertensive**	**normotensive**	**tissue**	**DEG**	**log2₂**	P_{ADJ}	Ref
**i**	**ii**	**iii**	**iv**	**v**	**vi**	**vii**	**viii**	**ix**	**x**	**xi**	xii
96	*Pla2g5*	3.85	0.05	rat	ISIAH	WAG	adrenal gland	*Pla2g2d*	2.49	10^{-2}	[47]
97	*Pla2g5*	3.85	0.05	rat	SHR	Wistar	brain pericytes	*Pla2g2a*	4.74	0.05	[48]
98	*Pla2g5*	3.85	0.05	rat	ISIAH	WAG	adrenal gland	*Pla2g16*	0.62	10^{-2}	[47]
99	*Pla2g5*	3.85	0.05	rat	ISIAH	WAG	adrenal gland	*Pla2g7*	−0.97	10^{-2}	[47]
100	*Pla2g5*	3.85	0.05	rat	ISIAH	WAG	brain stem	*Pla2g3*	−0.84	10^{-2}	[43]
101	*Pla2g5*	3.85	0.05	rat	ISIAH	WAG	brain stem	*Pld5*	−0.99	10^{-2}	[43]
102	*Pla2g5*	3.85	0.05	rat	ISIAH	WAG	hypothalamus	*Pld5*	−0.77	10^{-2}	[44]
103	*Pla2g5*	3.85	0.05	rat	ISIAH	WAG	renal medulla	*Pla2g4a*	−0.69	10^{-2}	[45]
104	*Pla2g5*	3.85	0.05	rat	ISIAH	WAG	renal medulla	*Pld6*	0.87	0.05	[45]
105	*Pla2g5*	3.85	0.05	rat	ISIAH	WAG	renal cortex	*Pla2g16*	0.58	0.05	[46]
106	*Pla2g5*	3.85	0.05	rat	ISIAH	WAG	renal cortex	*Pla2g7*	0.71	0.05	[46]
107	*Pla2g5*	3.85	0.05	rat	OXIS	Wistar	retina	*Plaat1*	−2.28	10^{-7}	[42]
108	*Pla2g5*	3.85	0.05	rat	OXIS	Wistar	hippocampus	*Pla2g6*	0.93	10^{-5}	[40]
109	*Pla2g5*	3.85	0.05	rat	OXIS	Wistar	hippocampus	*Plcg2*	−0.61	10^{-2}	[40]
110	*Pla2g5*	3.85	0.05	rabbit	Goldblatt 2-kidney 1-clip exposed	norm	middle cerebral artery	*Pla2g1b*	1.26	10^{-4}	[55]
111	*Pla2g5*	3.85	0.05	mice	BPH/2J	BPN/3J	kidney	*Pla2g5*	2.59	10^{-6}	[54]
112	*Plod1*	−0.67	0.05	rat	ISIAH	WAG	hypothalamus	*Plod3*	1.14	0.05	[44]
113	*Plod1*	−0.67	0.05	rat	ISIAH	WAG	adrenal gland	*Plod2*	−0.98	10^{-2}	[47]
114	*Plod1*	−0.67	0.05	mice	BPH/2J	BPN/3J	kidney	*Plod2*	1.53	10^{-3}	[54]
115	*Ppp1r3b*	2.45	0.05	rat	ISIAH	WAG	renal medulla	*Ppp1r3b*	−0.94	10^{-2}	[45]
116	*Ppp1r3b*	2.45	0.05	rat	ISIAH	WAG	adrenal gland	*Ppp1r3b*	1.21	10^{-2}	[47]
117	*Ppp1r3b*	2.45	0.05	rat	OXYS	Wistar	prefrontal cortex	*Ppp1r3b*	0.74	10^{-2}	[41]
118	*Ppp1r3b*	2.45	0.05	rat	OXIS	Wistar	hippocampus	*Ppp3r1*	−0.37	10^{-3}	[40]
119	*Prlr*	6.43	10^{-2}	mice	BPH/2J	BPN/3J	kidney	*Prlr*	2.12	10^{-2}	[54]
120	*Rbm3*	0.89	0.05	rat	ISIAH	WAG	renal cortex	*Rbm3*	0.49	0.05	[46]
121	*Rbm3*	0.89	0.05	rat	OXYS	Wistar	prefrontal cortex	*Rbm3*	−0.35	0.05	[41]
122	*Rbm3*	0.89	0.05	rat	ISIAH	WAG	renal medulla	*Rbm20*	0.96	10^{-2}	[45]
123	*Rbm3*	0.89	0.05	rat	ISIAH	WAG	adrenal gland	*Rbm43*	0.80	10^{-2}	[47]
124	*Rbm3*	0.89	0.05	mice	BPH/2J	BPN/3J	kidney	*Rbm10*	−1.25	0.05	[54]
125	*Rbm3*	0.89	0.05	mice	BPH/2J	BPN/3J	kidney	*Rbm39*	−1.61	0.05	[54]
126	*Rbm3*	0.89	0.05	mice	BPH/2J	BPN/3J	kidney	*Rbm4b*	−1.61	10^{-4}	[54]
127	*Rbm3*	0.89	0.05	rabbit	Goldblatt 2-kidney 1-clip exposed	norm	middle cerebral artery	*Rbm12b*	2.00	10^{-4}	[55]

Table S1. *Cont.*

#	hippocampus, tame *vs* aggressive rats			DEGs in the tissues of the hypertensive *vs* normotensive animals as said by others that we could find							
	DEG	**log2$_2$**	P$_{ADJ}$	**species**	**hypertensive**	**normotensive**	**tissue**	***DEG***	**log2$_2$**	P$_{ADJ}$	Ref
**i**	**ii**	**iii**	**iv**	**v**	**vi**	**vii**	**viii**	**ix**	**x**	**xi**	xii
128	*Retsat*	−0.98	0.05	rat	ISIAH	WAG	brain stem	*Retsat*	1.86	10^{-2}	[43]
129	*Retsat*	−0.98	0.05	rat	ISIAH	WAG	hypothalamus	*Retsat*	1.82	10^{-2}	[44]
130	*Retsat*	−0.98	0.05	rat	ISIAH	WAG	renal medulla	*Retsat*	2.05	10^{-2}	[45]
131	*Retsat*	−0.98	0.05	rat	ISIAH	WAG	renal cortex	*Retsat*	1.85	0.05	[46]
132	*Retsat*	−0.98	0.05	rat	ISIAH	WAG	adrenal gland	*Retsat*	2.59	10^{-2}	[47]
133	*Retsat*	−0.98	0.05	rat	OXIS	Wistar	retina	*Retsat*	−0.97	0.05	[42]
134	*Slc16a12*	3.08	0.05	rat	ISIAH	WAG	adrenal gland	*Slc16a12*	0.91	10^{-2}	[47]
135	*Slc16a12*	3.08	0.05	rat	ISIAH	WAG	adrenal gland	*Slc16a14*	−1.86	10^{-2}	[47]
136	*Slc16a12*	3.08	0.05	rat	ISIAH	WAG	adrenal gland	*Slc16a6*	0.95	10^{-2}	[47]
137	*Slc16a12*	3.08	0.05	rat	ISIAH	WAG	renal medulla	*Slc16a1*	0.51	0.05	[45]
138	*Slc16a12*	3.08	0.05	rat	ISIAH	WAG	renal medulla	*Slc16a14*	1.14	10^{-2}	[45]
139	*Slc4a5*	6.27	10^{-3}	rat	ISIAH	WAG	renal medulla	*Slc4a11*	−0.87	10^{-2}	[45]
140	*Slc4a5*	6.27	10^{-3}	rat	ISIAH	WAG	renal medulla	*Slc4a3*	−0.84	10^{-2}	[45]
141	*Slc4a5*	6.27	10^{-3}	rat	ISIAH	WAG	renal medulla	*Slc4a7*	−0.79	10^{-2}	[45]
142	*Slc4a5*	6.27	10^{-3}	rat	ISIAH	WAG	renal cortex	*Slc4a1*	0.86	0.05	[46]
143	*Slc4a5*	6.27	10^{-3}	rat	ISIAH	WAG	renal cortex	*Slc4a9*	0.63	0.05	[46]
144	*Slc4a5*	6.27	10^{-3}	rat	ISIAH	WAG	adrenal gland	*Slc4a11*	−1.83	10^{-2}	[47]
145	*Slc4a5*	6.27	10^{-3}	rat	ISIAH	WAG	adrenal gland	*Slc4a3*	−0.54	0.05	[47]
146	*Slc4a5*	6.27	10^{-3}	rat	ISIAH	WAG	adrenal gland	*Slc4a4*	0.96	0.05	[47]
147	*Slc4a5*	6.27	10^{-3}	mice	BPH/2J	BPN/3J	kidney	*Slc4a1*	1.47	10^{-3}	[54]
148	*Smoc2*	−2.09	0.05	rat	ISIAH	WAG	adrenal gland	*Smoc2*	1.12	10^{-2}	[47]
149	*Smoc2*	−2.09	0.05	rat	ISIAH	WAG	renal medulla	*Smoc1*	−0.90	0.05	[45]
150	*Tc2n*	3.47	10^{-2}	rat	ISIAH	WAG	renal medulla	*Tc2n*	−0.83	0.05	[45]
151	*Ucp2*	0.73	0.05	rat	ISIAH	WAG	adrenal gland	*Ucp1*	8.89	10^{-2}	[47]

Notes. Hereinafter, log2: the log_2-transformed fold change (i.e., a ratio of an expression level of a given gene in tame rats to that in aggressive rats); P$_{ADJ}$: statistical significance according to Fisher's Z-test with the Benjamini correction for multiple comparisons, respectively. ISIAH, OXYS, SD, SHR, WAG, Wistar, BPH/2J (inbred) and BPN/3J (inbred) as laboratory rat lines; Genes: *Aqp1*, aquaporin 1; *Ascl3*, achaete-scute family BHLH transcription factor 3; *Cckbr*, cholecystokinin B receptor; *Defb17*, defensin β17; *Enpp2*, ectonucleotide pyrophosphatase / phosphodiesterase 2; *Frem1*, Fras1-related extracellular matrix 1; *Gpd1*, glycerol-3-phosphate dehydrogenase 1; *Hbb-b1*, hemoglobin β adult major chain; *Htr2c*, 5-hydroxytryptamine (serotonin) receptor 2C; *Krt2*, keratin 2; *Lilrb3l*, leukocyte immunoglobulin-like receptor, subfamily B, member 3-like; *Lypd1*, Ly6/Plaur domain containing 1; *Morn1*, MORN repeat containing 1; *Myom2*, myomesin 2; *Pcdhb9*, protocadherin β9; *Pla2g2d*, phospholipase A2, group IID; *Pla2g5*, phospholipase A2, group V; *Plod1*, procollagen-lysine, 2-oxoglutarate 5-dioxygenase 1; *Ppp1r3b*, protein phosphatase 1, regulatory subunit 3B; *Prlr*; prolactin receptor; *Rbm3*, RNA binding motif protein 3; *Retsat*, retinol saturase; Slc16a12, solute carrier family 16, member 12; *Slc4a5*, solute carrier family 4 member 5; *Smoc2*, SPARC related modular calcium binding 2; *Tc2n*, tandem C2 domains, nuclear; *Ucp2*, uncoupling protein 2.

Table S2. The tame-versus-aggressive-rat hippocampal DEGs found in this work in comparison with their known homologous DEGs—in hypertensive versus normotensive patients as reported by others—that we could find.

#	hippocampus, tame *vs* aggressive rats			DEGs in the tissues of the hypertensive *vs* normotensive patients as said by others that we could find						
	DEG	**log2$_2$**	P_{ADJ}	**hypertensive**	**normotensive**	**tissue**	***DEG***	**log2$_2$**	P_{ADJ}	Ref
**i**	**ii**	**iii**	**iv**	**v**	**vi**	**vii**	**viii**	**ix**	**x**	**xi**
1	*Alb*	3.21	10^{-7}	idiopathic pulmonary hypertension	norm	lung	*ALB*	−0.93	0.05	[27]
2	*Alb*	3.21	10^{-7}	pulmonary hypertension at fibrosis	norm	lung	*ALB*	1.58	10^{-2}	[30]
3	*Aqp1*	5.91	10^{-2}	preeclampsia	norm	placenta	*AQP1*	0.18	10^{-2}	[32]
4	*Aqp1*	5.91	10^{-2}	preeclampsia	norm	placenta	*AQP3*	0.25	10^{-3}	[32]
5	*Aqp1*	5.91	10^{-2}	pulmonary hypertension at fibrosis	norm	lung	*AQP10*	−1.77	10^{-2}	[30]
6	*Aqp1*	5.91	10^{-2}	HT-caused coronary artery disease	norm	peripheral blood	*AQP9*	2.42	0.05	[39]
7	*Ascl3*	2.38	0.05	pulmonary hypertension at fibrosis	norm	lung	*ASCL2*	−1.46	10^{-3}	[30]
8	*Bag3*	−0.92	0.05	pulmonary hypertension at fibrosis	norm	lung	*BAG4*	−1.02	10^{-2}	[30]
9	*Bag3*	−0.92	0.05	pulmonary hypertension at fibrosis	norm	lung	*BAG5*	0.93	10^{-2}	[30]
10	*Bag3*	−0.92	0.05	pulmonary hypertension at fibrosis	norm	lung	*BAG6*	−0.75	10^{-2}	[30]
11	*Bag3*	−0.92	0.05	HT-caused coronary artery disease	norm	peripheral blood	*BAG1*	3.86	0.05	[39]
12	*Baiap2l1*	3.67	0.05	pulmonary hypertension at fibrosis	norm	lung	*BAIAP2*	−0.98	10^{-2}	[30]
13	*Bdh1*	0.40	0.05	pulmonary hypertension at fibrosis	norm	lung	*BDH1*	−0.97	10^{-2}	[30]
14	*Bdh1*	0.40	0.05	HT-caused coronary artery disease	norm	peripheral blood	*BDH2*	−1.10	0.05	[39]
15	*Defb17*	5.94	0.05	preeclampsia	norm	placenta	*DEFA4*	−0.22	10^{-2}	[32]
16	*Defb17*	5.94	0.05	pulmonary hypertension at fibrosis	norm	lung	*DEFB104B*	−1.35	10^{-3}	[30]
17	*Defb17*	5.94	0.05	HT-caused coronary artery disease	norm	peripheral blood	*DEFA4*	2.14	0.05	[39]
18	*Enpp2*	2.41	0.05	preeclampsia	norm	placenta	*ENPP2*	−0.22	0.05	[32]
19	*Enpp2*	2.41	0.05	BMPR2-deficient endothelial cells	norm	pulmonary artery	*ENPP2*	−3.22	10^{-5}	[31]
20	*Enpp2*	2.41	0.05	HT-caused atrial fibrillation	norm	auricle tissue biopsy	*ENPP2*	1.25	10^{-2}	[39]
21	*Enpp2*	2.41	0.05	HT-caused coronary artery disease	norm	peripheral blood	*ENPP7*	1.99	0.05	[39]
22	*Frem1*	3.16	0.05	pulmonary hypertension at fibrosis	norm	lung	*FREM3*	1.67	10^{-2}	[30]
23	*Gpd1*	−1.34	10^{-3}	pulmonary hypertension at fibrosis	norm	lung	*GPD1*	−1.98	10^{-2}	[30]
24	*Gpd1*	−1.34	10^{-3}	preeclampsia	norm	placenta	*GPD1L*	0.11	0.05	[32]
25	*Gpd1*	−1.34	10^{-3}	preeclampsia	norm	decidua basalis	*GPD1L*	0.20	0.05	[35]
26	*Hbb-b1*	−6.19	10^{-4}	preeclampsia	norm	placenta	*HBD*	−0.63	10^{-3}	[32]
27	*Hbb-b1*	−6.19	10^{-4}	pulmonary hypertension at fibrosis	norm	lung	*HBD*	−2.83	10^{-3}	[30]
28	*Hbb-b1*	−6.19	10^{-4}	pulmonary arterial hypertension	norm	lung	*HBA1*	2.08	10^{-9}	[28]
29	*Hbb-b1*	−6.19	10^{-4}	pulmonary arterial hypertension	norm	lung	*HBB*	2.46	10^{-10}	[28]
30	*Hbb-b1*	−6.19	10^{-4}	HT-caused coronary artery disease	norm	peripheral blood	*HBBP1*	1.03	0.05	[39]
31	*Hbb-b1*	−6.19	10^{-4}	HT-caused coronary artery disease	norm	peripheral blood	*HBE1*	1.42	0.05	[39]
32	*Hbb-b1*	−6.19	10^{-4}	HT-caused coronary artery disease	norm	peripheral blood	*HBG2(1)*	4.49	0.05	[39]

Table S2. *Cont*

#	hippocampus, tame *vs* aggressive rats			DEGs in the tissues of the hypertensive *vs* normotensive patients as said by others that we could find						
	DEG	**log2**$_2$	P_{ADJ}	**hypertensive**	**normotensive**	**tissue**	***DEG***	**log2**$_2$	P_{ADJ}	Ref
**i**	**ii**	**iii**	**iv**	**v**	**vi**	**vii**	**viii**	**ix**	**x**	**xi**
33	*Hbb-b1*	−6.19	10^{-4}	HT-caused coronary artery disease	norm	peripheral blood	*HBM*	5.33	0.05	[39]
34	*Hbb-b1*	−6.19	10^{-4}	HT-caused coronary artery disease	norm	peripheral blood	*HBQ1*	3.10	0.05	[39]
35	*Hbb-b1*	−6.19	10^{-4}	HT-caused atrial fibrillation	norm	auricle tissue biopsy	*HBA2(1)*	2.37	10^{-2}	[39]
36	*Htr2c*	2.03	0.05	preeclampsia	norm	placenta	*HTR3C*	0.14	0.05	[32]
37	*Htr2c*	2.03	0.05	pulmonary hypertension at fibrosis	norm	lung	*HTR1B*	−1.22	10^{-2}	[30]
38	*Htr2c*	2.03	0.05	pulmonary hypertension at fibrosis	norm	lung	*HTR7*	0.85	10^{-2}	[30]
39	*Htr2c*	2.03	0.05	BMPR2-deficient endothelial cells	norm	pulmonary artery	*HTR2B*	3.32	10^{-3}	[31]
40	*Krt2*	−1.43	10^{-3}	preeclampsia	norm	placenta	*KRT19*	0.35	10^{-3}	[32]
41	*Krt2*	−1.43	10^{-3}	pulmonary hypertension at fibrosis	norm	lung	*KRT32*	−0.95	10^{-2}	[30]
42	*Krt2*	−1.43	10^{-3}	pulmonary hypertension at fibrosis	norm	lung	*KRT33A*	−1.25	10^{-3}	[30]
43	*Krt2*	−1.43	10^{-3}	pulmonary hypertension at fibrosis	norm	lung	*KRT33B*	−0.71	10^{-2}	[30]
44	*Krt2*	−1.43	10^{-3}	pulmonary hypertension at fibrosis	norm	lung	*KRT36*	−0.89	10^{-2}	[30]
45	*Krt2*	−1.43	10^{-3}	pulmonary hypertension at fibrosis	norm	lung	*KRT76*	−1.58	10^{-2}	[30]
46	*Krt2*	−1.43	10^{-3}	pulmonary hypertension at fibrosis	norm	lung	*KRT79*	−1.25	10^{-2}	[30]
47	*Krt2*	−1.43	10^{-3}	pulmonary hypertension at fibrosis	norm	lung	*KRT9*	−1.35	10^{-2}	[30]
48	*Krt2*	−1.43	10^{-3}	BMPR2-deficient endothelial cells	norm	pulmonary artery	*KRT19*	3.82	10^{-3}	[31]
49	*Krt2*	−1.43	10^{-3}	BMPR2-deficient endothelial cells	norm	pulmonary artery	*KRT7*	9.02	10^{-6}	[31]
50	*Krt2*	−1.43	10^{-3}	HT-caused coronary artery disease	norm	peripheral blood	*KRT1*	2.28	10^{-2}	[39]
51	*Krt2*	−1.43	10^{-3}	HT-caused coronary artery disease	norm	peripheral blood	*KRT10*	−1.13	0.05	[39]
52	*Krt2*	−1.43	10^{-3}	HT-caused coronary artery disease	norm	peripheral blood	*KRT23*	2.64	0.05	[39]
53	*Krt2*	−1.43	10^{-3}	HT-caused coronary artery disease	norm	peripheral blood	*KRT33B*	1.10	0.05	[39]
54	*Krt2*	−1.43	10^{-3}	severe preeclampsia	norm	blood	*KRT72*	1.20	0.05	[34]
55	*Krt2*	−1.43	10^{-3}	HT-caused squamous cancer	norm	squamous lung tumor	*KRT13*	5.20	10^{-2}	[38]
56	*Krt2*	−1.43	10^{-3}	HT-caused squamous cancer	norm	squamous lung tumor	*KRT16*	3.17	0.05	[38]
57	*Krt2*	−1.43	10^{-3}	HT-caused squamous cancer	norm	squamous lung tumor	*KRT5*	1.88	0.05	[38]
58	*Krt2*	−1.43	10^{-3}	HT-caused squamous cancer	norm	squamous lung tumor	*KRT6B*	6.99	0.05	[38]
59	*Krt2*	−1.43	10^{-3}	HT-caused squamous cancer	norm	squamous lung tumor	*KRT6C*	3.95	0.05	[38]
60	*Krt2*	−1.43	10^{-3}	preeclampsia	norm	decidua basalis	*KRT86*	0.42	0.05	[35]
61	*Lilrb3l*	7.45	0.05	preeclampsia	norm	placenta	*LILRA2*	−0.22	0.05	[32]
62	*Lilrb3l*	7.45	0.05	preeclampsia	norm	placenta	*LILRA3*	−0.15	0.05	[32]
63	*Lilrb3l*	7.45	0.05	preeclampsia	norm	placenta	*LILRB1*	−0.14	10^{-3}	[32]

Table S2. *Cont*

#	hippocampus, tame *vs* aggressive rats			DEGs in the tissues of the hypertensive *vs* normotensive patients as said by others that we could find						
	DEG	**log2₂**	P_{ADJ}	**hypertensive**	**normotensive**	**tissue**	***DEG***	**log2₂**	P_{ADJ}	Ref
**i**	**ii**	**iii**	**iv**	**v**	**vi**	**vii**	**viii**	**ix**	**x**	**xi**
64	*Lilrb3l*	7.45	0.05	pulmonary hypertension at fibrosis	norm	lung	*LILRB2*	0.91	10^{-3}	[30]
65	*Lilrb3l*	7.45	0.05	pulmonary hypertension at fibrosis	norm	lung	*LILRB4*	−1.01	10^{-2}	[30]
66	*Lilrb3l*	7.45	0.05	female-specific pulmonary HT	norm	lung	*LILRB3*	−1.05	10^{-4}	[29]
67	*Lilrb3l*	7.45	0.05	preeclampsia	norm	decidua basalis	*LILRB1*	−0.16	0.05	[35]
68	*Lilrb3l*	7.45	0.05	preeclampsia	norm	decidua basalis	*LILRB3*	−1.17	0.05	[35]
69	*Lypd1*	−0.89	0.05	BMPR2-deficient endothelial cells	norm	pulmonary artery	*LYPD1*	3.03	10^{-7}	[31]
70	*Lypd1*	−0.89	0.05	BMPR2-deficient endothelial cells	norm	pulmonary artery	*LY6K*	2.37	10^{-3}	[31]
71	*Lypd1*	−0.89	0.05	pulmonary hypertension at fibrosis	norm	lung	*LY6G6C*	−1.07	10^{-4}	[30]
72	*Lypd1*	−0.89	0.05	pulmonary hypertension at fibrosis	norm	lung	*LY6G6F*	−2.28	10^{-4}	[30]
73	*Lypd1*	−0.89	0.05	pulmonary hypertension at fibrosis	norm	lung	*LYPD3*	1.49	10^{-2}	[30]
74	*Morn1*	1.42	10^{-3}	HT-caused coronary artery disease	norm	peripheral blood	*MORN1*	1.09	10^{-2}	[39]
75	*Pcdhb9*	−1.03	0.05	pulmonary hypertension at fibrosis	norm	lung	*PCDHB10*	1.89	10^{-2}	[30]
76	*Pcdhb9*	−1.03	0.05	pulmonary hypertension at fibrosis	norm	lung	*PCDHB15*	1.47	10^{-4}	[30]
77	*Pcdhb9*	−1.03	0.05	pulmonary hypertension at fibrosis	norm	lung	*PCDHB16*	1.38	10^{-4}	[30]
78	*Pcdhb9*	−1.03	0.05	pulmonary hypertension at fibrosis	norm	lung	*PCDHB17P*	1.21	10^{-2}	[30]
79	*Pcdhb9*	−1.03	0.05	pulmonary hypertension at fibrosis	norm	lung	*PCDHB4*	2.93	10^{-4}	[30]
80	*Pcdhb9*	−1.03	0.05	pulmonary hypertension at fibrosis	norm	lung	*PCDHB6*	1.35	10^{-2}	[30]
81	*Pcdhb9*	−1.03	0.05	HT-caused coronary artery disease	norm	peripheral blood	*PCDHB11*	1.12	0.05	[39]
82	*Pcdhb9*	−1.03	0.05	HT-caused coronary artery disease	norm	peripheral blood	*PCDHB13*	1.04	0.05	[39]
83	*Pcdhga1*	2.45	0.05	pulmonary hypertension at fibrosis	norm	lung	*PCDHGB7*	1.22	10^{-2}	[30]
84	*Pcdhga1*	2.45	0.05	preeclampsia	norm	decidua basalis	*GPD1L*	−0.05	0.05	[35]
85	*Pla2g2d*	2.84	0.05	pulmonary hypertension at fibrosis	norm	lung	*PLA2G2D*	−1.06	10^{-2}	[30]
86	*Pla2g2d*	2.84	0.05	pulmonary hypertension at fibrosis	norm	lung	*PLA2G10*	−1.26	10^{-2}	[30]
87	*Pla2g2d*	2.84	0.05	pulmonary hypertension at fibrosis	norm	lung	*PLA2G12A*	−0.67	10^{-2}	[30]
88	*Pla2g2d*	2.84	0.05	pulmonary hypertension at fibrosis	norm	lung	*PLA2G12B*	−2.27	10^{-4}	[30]
89	*Pla2g2d*	2.84	0.05	pulmonary hypertension at fibrosis	norm	lung	*PLA2G15*	−0.95	10^{-2}	[30]
90	*Pla2g2d*	2.84	0.05	pulmonary hypertension at fibrosis	norm	lung	*PLA2G1B*	−1.74	10^{-2}	[30]
91	*Pla2g2d*	2.84	0.05	pulmonary hypertension at fibrosis	norm	lung	*PLA2G2F*	−1.20	10^{-2}	[30]
92	*Pla2g2d*	2.84	0.05	pulmonary hypertension at fibrosis	norm	lung	*PLA2G3*	−1.46	10^{-2}	[30]
93	*Pla2g2d*	2.84	0.05	pulmonary hypertension at fibrosis	norm	lung	*PLA2G4F*	−1.54	10^{-3}	[30]
94	*Pla2g2d*	2.84	0.05	preeclampsia	norm	placenta	*PLA2G16*	0.25	10^{-4}	[32]

Table S2. *Cont*

#	hippocampus, tame *vs* aggressive rats			DEGs in the tissues of the hypertensive *vs* normotensive patients as said by others that we could find						
	DEG	**log2₂**	P_{ADJ}	**hypertensive**	**normotensive**	**tissue**	***DEG***	**log2₂**	P_{ADJ}	Ref
**i**	**ii**	**iii**	**iv**	**v**	**vi**	**vii**	**viii**	**ix**	**x**	**xi**
95	*Pla2g2d*	2.84	0.05	BMPR2-deficient endothelial cells	norm	pulmonary artery	*PLA2G16*	2.11	10^{-7}	[31]
96	*Pla2g2d*	2.84	0.05	BMPR2-deficient endothelial cells	norm	pulmonary artery	*PLA2G4C*	−2.30	10^{-4}	[31]
97	*Pla2g2d*	2.84	0.05	HT-caused coronary artery disease	norm	peripheral blood	*PLA2G12A*	−1.31	0.05	[39]
98	*Pla2g2d*	2.84	0.05	HT-caused coronary artery disease	norm	peripheral blood	*PLA2G7*	−1.18	0.05	[39]
99	*Pla2g2d*	2.84	0.05	HT-caused atrial fibrillation	norm	auricle tissue biopsy	*PLA2G12B*	−1.29	10^{-2}	[39]
100	*Pla2g5*	3.85	0.05	pulmonary hypertension at fibrosis	norm	lung	*PLA2G2D*	−1.06	10^{-2}	[30]
101	*Pla2g5*	3.85	0.05	pulmonary hypertension at fibrosis	norm	lung	*PLA2G10*	−1.26	10^{-2}	[30]
102	*Pla2g5*	3.85	0.05	pulmonary hypertension at fibrosis	norm	lung	*PLA2G12A*	−0.67	10^{-2}	[30]
103	*Pla2g5*	3.85	0.05	pulmonary hypertension at fibrosis	norm	lung	*PLA2G12B*	−2.27	10^{-4}	[30]
104	*Pla2g5*	3.85	0.05	pulmonary hypertension at fibrosis	norm	lung	*PLA2G15*	−0.95	10^{-2}	[30]
105	*Pla2g5*	3.85	0.05	pulmonary hypertension at fibrosis	norm	lung	*PLA2G1B*	−1.74	10^{-2}	[30]
106	*Pla2g5*	3.85	0.05	pulmonary hypertension at fibrosis	norm	lung	*PLA2G2F*	−1.20	10^{-2}	[30]
107	*Pla2g5*	3.85	0.05	pulmonary hypertension at fibrosis	norm	lung	*PLA2G3*	−1.46	10^{-2}	[30]
108	*Pla2g5*	3.85	0.05	pulmonary hypertension at fibrosis	norm	lung	*PLA2G4F*	−1.54	10^{-3}	[30]
109	*Pla2g5*	3.85	0.05	preeclampsia	norm	placenta	*PLA2G16*	0.25	10^{-4}	[32]
110	*Pla2g5*	3.85	0.05	BMPR2-deficient endothelial cells	norm	pulmonary artery	*PLA2G16*	2.11	10^{-7}	[31]
111	*Pla2g5*	3.85	0.05	BMPR2-deficient endothelial cells	norm	pulmonary artery	*PLA2G4C*	−2.30	10^{-4}	[31]
112	*Pla2g5*	3.85	0.05	HT-caused coronary artery disease	norm	peripheral blood	*PLA2G12A*	−1.31	0.05	[39]
113	*Pla2g5*	3.85	0.05	HT-caused coronary artery disease	norm	peripheral blood	*PLA2G7*	−1.18	0.05	[39]
114	*Pla2g5*	3.85	0.05	HT-caused atrial fibrillation	norm	auricle tissue biopsy	*PLA2G12B*	−1.29	10^{-2}	[39]
115	*Plod1*	−0.67	0.05	preeclampsia	norm	placenta	*PLOD2*	0.18	10^{-2}	[32]
116	*Ppp1r3b*	2.45	0.05	pulmonary hypertension at fibrosis	norm	lung	*PPP1R3B*	1.92	10^{-3}	[30]
117	*Pygl*	−1.21	10^{-2}	pulmonary hypertension at fibrosis	norm	lung	*PYGB*	−0.82	10^{-2}	[30]
118	*Rbm3*	0.89	0.05	pulmonary hypertension at fibrosis	norm	lung	*RBM3*	0.93	10^{-2}	[30]
119	*Rbm3*	0.89	0.05	pulmonary hypertension at fibrosis	norm	lung	*RBM15*	−1.17	10^{-2}	[30]
120	*Rbm3*	0.89	0.05	pulmonary hypertension at fibrosis	norm	lung	*RBM17*	0.96	10^{-2}	[30]
121	*Rbm3*	0.89	0.05	pulmonary hypertension at fibrosis	norm	lung	*RBM20*	1.99	10^{-2}	[30]
122	*Rbm3*	0.89	0.05	pulmonary hypertension at fibrosis	norm	lung	*RBM34*	0.80	10^{-2}	[30]
123	*Rbm3*	0.89	0.05	pulmonary hypertension at fibrosis	norm	lung	*RBM48*	0.55	10^{-2}	[30]
124	*Rbm3*	0.89	0.05	pulmonary hypertension at fibrosis	norm	lung	*RBM8A*	1.79	10^{-2}	[30]
125	*Rbm3*	0.89	0.05	pulmonary hypertension at fibrosis	norm	lung	*RBMX2*	0.62	10^{-2}	[30]

Table S2. *Cont*

#	hippocampus, tame *vs* aggressive rats			DEGs in the tissues of the hypertensive *vs* normotensive patients as said by others that we could find						
	DEG	**log2**$_2$	P_{ADJ}	**hypertensive**	**normotensive**	**tissue**	***DEG***	**log2**$_2$	P_{ADJ}	Ref
**i**	**ii**	**iii**	**iv**	**v**	**vi**	**vii**	**viii**	**ix**	**x**	**xi**
126	*Rbm3*	0.89	0.05	preeclampsia	norm	placenta	*RBM2B*	0.07	10^{-2}	[32]
127	*Rbm3*	0.89	0.05	preeclampsia	norm	placenta	*RBM43*	−0.07	0.05	[32]
128	*Rbm3*	0.89	0.05	preeclampsia	norm	placenta	*RBMX2*	−0.05	0.05	[32]
129	*Rbm3*	0.89	0.05	BMPR2-deficient endothelial cells	norm	pulmonary artery	*RBM24*	−2.68	10^{-3}	[31]
130	*Rbm3*	0.89	0.05	HT-caused coronary artery disease	norm	peripheral blood	*RBM15*	−1.97	0.05	[39]
131	*Rbm3*	0.89	0.05	HT-caused coronary artery disease	norm	peripheral blood	*RBM26*	−2.58	0.05	[39]
132	*Rbm3*	0.89	0.05	HT-caused coronary artery disease	norm	peripheral blood	*RBM33*	−1.46	0.05	[39]
133	*Rbm3*	0.89	0.05	HT-caused coronary artery disease	norm	peripheral blood	*RBM39*	−2.13	0.05	[39]
134	*Rbm3*	0.89	0.05	HT-caused coronary artery disease	norm	peripheral blood	*RBM4*	−2.14	10^{-2}	[39]
135	*Rbm3*	0.89	0.05	HT-caused coronary artery disease	norm	peripheral blood	*RBM41*	−1.72	10^{-2}	[39]
136	*Rbm3*	0.89	0.05	HT-caused coronary artery disease	norm	peripheral blood	*RBM6*	−2.27	0.05	[39]
137	*Retsat*	−0.98	0.05	pulmonary hypertension at fibrosis	norm	lung	*RETSAT*	−0.83	10^{-3}	[30]
138	*Slc16a12*	3.08	0.05	pulmonary hypertension at fibrosis	norm	lung	*SLC16A12*	2.50	10^{-2}	[30]
139	*Slc16a12*	3.08	0.05	preeclampsia	norm	placenta	*SLC16A10*	−0.14	0.05	[32]
140	*Slc16a12*	3.08	0.05	preeclampsia	norm	placenta	*SLC16A2*	−0.11	0.05	[32]
141	*Slc16a12*	3.08	0.05	preeclampsia	norm	placenta	*SLC16A4*	−0.16	0.05	[32]
142	*Slc16a12*	3.08	0.05	pulmonary hypertension at fibrosis	norm	lung	*SLC16A11*	−1.14	10^{-3}	[30]
143	*Slc16a12*	3.08	0.05	pulmonary hypertension at fibrosis	norm	lung	*SLC16A14*	1.69	10^{-2}	[30]
144	*Slc16a12*	3.08	0.05	HT-caused coronary artery disease	norm	peripheral blood	*SLC16A10*	−2.37	10^{-2}	[39]
145	*Slc16a12*	3.08	0.05	HT-caused coronary artery disease	norm	peripheral blood	*SLC16A7*	−1.19	0.05	[39]
146	*Slc4a5*	6.27	10^{-3}	pulmonary hypertension at fibrosis	norm	lung	*SLC4A1*	−3.05	10^{-2}	[30]
147	*Slc4a5*	6.27	10^{-3}	HT-caused coronary artery disease	norm	peripheral blood	*SLC4A1*	3.48	0.05	[39]
148	*Slc4a5*	6.27	10^{-3}	HT-caused coronary artery disease	norm	peripheral blood	*SLC4A7*	−1.38	0.05	[39]
149	*Smoc2*	−2.09	0.05	pulmonary hypertension at fibrosis	norm	lung	*SMOC2*	1.83	10^{-2}	[30]
150	*Smoc2*	−2.09	0.05	HT-caused atrial fibrillation	norm	auricle tissue biopsy	*SMOC2*	1.29	10^{-3}	[39]
151	*Spint1*	−1.39	10^{-4}	pulmonary hypertension at fibrosis	norm	lung	*SPINT1*	−0.82	10^{-2}	[30]
152	*Spint1*	−1.39	10^{-4}	preeclampsia	norm	placenta	*SPINT2*	0.19	10^{-2}	[32]
153	*Tc2n*	3.47	10^{-2}	HT-caused coronary artery disease	norm	peripheral blood	*TC2N*	−1.21	0.05	[39]
154	*Tecta*	1.38	10^{-5}	HT-caused coronary artery disease	norm	peripheral blood	*TECTA*	1.25	10^{-2}	[39]
155	*Txnrd2*	−0.71	10^{-2}	pulmonary hypertension at fibrosis	norm	lung	*TXNRD2*	1.26	10^{-2}	[30]
156	*Txnrd2*	−0.71	10^{-2}	pulmonary hypertension at fibrosis	norm	lung	*TXNRD1*	1.69	10^{-2}	[30]
157	*Ucp2*	0.73	0.05	pulmonary hypertension at fibrosis	norm	lung	*UCP3*	−3.21	10^{-2}	[30]

Notes: see Notes under Table S2. HT, hypertension. Genes: *Alb*, albumin; *Bag3*, BAG cochaperone 3; *Baiap2l1, BAR/IMD domain containing adaptor protein 2 like 1; Bdh1, 3-hydroxybutyrate dehydrogenase 1;* *Pcdhga1*, protocadherin gamma subfamily A, 1; *Pygl, glycogen phosphorylase L; Spint1, serine peptidase inhibitor, Kunitz type 1; Tecta, tectorin alpha; Txnrd2, thioredoxin reductase 2.*

Table S3. Effects—on hypertension—of underexpression or overexpression of the human genes homologous to the DEGs (in the hippocampus of tame versus aggressive rats) identified in this work according to the current state of the PubMed database [69].

No	**Human Gene**	**Effect of the human gene expression alterations on the hypertension development [Ref]**			
		**Downregulation (↓)**	HT	**Upregulation (↑)**	HT
**i**	**ii**	**iii**	**iv**	**v**	**vi**
1	*ALB*	within a clinical research: hypoalbuminemia can increase risk acute respiratory distress syndrome comorbid for pulmonary hypertension [78]	→	according to a drug safety review report: exogenous albumin is a drug against hepatic cirrhosis with portal hypertension [79]	←
2	*AQP1*	within a clinical report: aquaporin deficit caused by mutations in the type II BMP receptor (*BMPR2*) leads pulmonary hypertension [80]	→	within human disease models using mice: increased blood pressure in the right ventricle [81]	→
3	*ASCL3*	within a meta-analysis of cancer-related transcriptomes: ASCL3 deficit is reliably often in kidney cancer [82], when hypertension met up to 60% [13]	→	within a meta-analysis of cancer-related transcriptomes: ASCL3 excess marks breast cancer [82], risks of which rises with hypertension in obese, physically inactive, postmenopausal women and is reducible due to short-term aerobic activity [10]	→
4	*BAG3*	within a left ventricle tissue study: higher risks of heart failure in hypertension [83]	→	within a clinical report: in hypertensive patients with type 2 diabetes, BAG3 excess was found [84]	→
5	*BAIAP2L1*	within cellular models of human diseases: slowed hepatocellular carcinoma progression down [85], an predictor for the poor prognosis of which is hypertension [14]	←	within cohort-based clinical studies: higher risks of renal cell carcinomas [86] associated with hypertension [87]	→
6	*BDH1*	within human disease models using BDH1-knockdown cells: higher risks of acute myeloid leukemia [88], which can masquerade as intracranial hypertension in children [9]	→	within human disease models using retroviral vector carrying BDH1 copy: lowed cancer cells proliferation in acute myeloid leukemia [88] miming pediatric intracranial hypertension [9]	←
7	*CCKBR*	within the human hypertensive nephropathy model using the CCKBR-knockout mice: aggravated renal fibrosis [16]	→	within human disease models using mice administered with angiotensin II: higher risks of hypertensive nephropathy [16]	→
8	*CSPG4*	within human disease models using dogs: anti-CSPG4 electrovaccination is a veterinary treatment against canine oral malignant melanoma [89], which is comorbid with hypertension [90]	←	within human disease models using canine: CSPG4-excess is a biomarker of melanoma [89], which is comorbid with hypertension [90]	→
9	*DEFB117*	according to a retrospective clinical review, higher risks of atopic dermatitis [91], which is comorbid with hypertension [18]	→	garlic extract carrying sulfur-containing amino acid S-1-propenyl cysteine lows hypertension [92] *via* synergy to human defensins, excess of which leads it too [93]	←
10	*ENPP2*	doxorubicin lows ENPP2 level causing cardiotoxicity [94] that leads to a violation of the heart's ability to adapt to hypertension in the elderly [95]	→	NPP2 excess prevents doxorubicin cardiotoxicity [94] to save the heart under hypertension [95] during anticancer chemotherapy with this drug in the elderly	←
11	*FREM1*	low FREM1 marks breast cancer [96], risks of which rises with hypertension in obese, physically inactive, postmenopausal women and is reducible due to short-term aerobic activity [10]	→	increased immune cell infiltration [97] as a marker of pulmonary hypertension in both patients and human disease models using mice [98]	→
12	*GPD1*	low GPD1 marks breast cancer [99], risks of which rises with hypertension in obese, physically inactive, postmenopausal women and is reducible due to short-term aerobic activity [10]	→	within ancient Indian medicine, curcumin upregulates GPD1 to overcome hyperosmotic stress caused by high-salt diets [100], which induces or exacerbates hypertension [101]	←
13	*HBD*	in hypertension ,thalassemia has vasculoprotective effects [102] due to reduced blood viscosity [198]	←	high-altitude environment provokes erythrocytosis, hyperhemoglobinemia and hypertension [103]	→
14	*HNF4A*	within human disease models using mice: higher risks of rheumatoid arthritis [104], which is comorbid with hypertension [105]	→	according to RNA-Seq data, higher risks of gastric cancer [106], surgical removal of which relieves hypertension [12]	→

Note: gene expression alteration: downregulation (↓) or upregulation (↑); ***HT***, as effects on the hypertension development: worsen (→) or relieve (←). ***Genes***:*ALB*, albumin; *AQP1*, aquaporin 1 (Colton blood group); *ASCL3*, achaete-scute family bhlh transcription factor 3; *BAG3*, BAG cochaperone 3; *BAIAP2L1*, BAR/IMD domain containing adaptor protein 2 like 1; *: BDH1*, 3-hydroxybutyrate dehydrogenase 1; *CCKBR*, cholecystokinin b receptor *CSPG4B*, chondroitin sulfate proteoglycan 4b; *DEFB17*, defensin beta 17; *ENPP2*, ectonucleotidepyrophosphatase/phosphodiesterase 2; *FREM1*, fras1 related extracellular matrix 1; *GPD1*, glycerol-3-phosphate dehydrogenase 1; *HBD*, hemoglobin, beta adult major chain; *HNF4A*, hepatocyte nuclear factor 4, alpha.

Table S3. *Cont.*

No	**Human Gene**	**Effect of the human gene expression alterations on the hypertension development [Ref]**			
		**Downregulation (↓)**	HT	**Upregulation (↑)**	HT
**i**	**ii**	**iii**	**iv**	**v**	**vi**
15	*HTR2C*	within the human disease models using Htr2c-knockout mice: susceptibility to seizure [107], which is comorbid with hypertension [19]	→	according to a pharmaceutical report: the HT2CR-agonist lorcaserin is a drug against obesity provoking hypertension [108]	←
16	*KRT2*	within human disease models using rats: higher risk of Parkinson's disease [109] when cognitive and motor functions are impaired in hypertension [110]	→	within a cohort proteomics study: KRT2-excess met among top 10 marker of viral myocarditis [111], COVID-19 related form of which was comorbid with only hypertension-caused interstitial fibrosis [112]	→
17	*LILRB3*	within human disease models using *Lilrb3*-knockout mice: increased lung histopathology in pulmonary hypertension [113]	→	LILRB3-excess maintain unique antigen-presenting properties of circulating myeloid dendritic cells in HIV-1-infected elite controllers [114], while HIV-1 proteins alter endothelial and smooth muscle functions towards hypertension [115]	←
18	*LYPD1*	within human disease models using rats: high risks myocardial infarction [116], a risk-factor of which is hypertension in the COVID-19 patients [117]	→	within human disease models using rats: low angiogenicity within heart [116], while hypertension is aggravated by increased angiogenesis [22]	←
19	*MORN1*	within human disease models using mice embryo lacking a MORN-repeat containing gene: cardiac arrest [118] comorbid with hypertension [119]	→	within the microarrays study of hypertension-comorbid coronary artery disease patients versus healthy peoples: MORN1 excess was a DEG [39]	→
20	*MYOM2*	within human disease models using rats administered with thyroid hormone: MYOM2 deficiency marks cardiac hypertrophy [120] comorbid with hypertension [121]	→	within the microarray of rats, who's hearts were subjected to ischemia comorbid with hypertension [122]: MYOM2 excess was found as a DEG [123]	→
21	*PCDHB9*	when treating prostate cancer, half doses of drugs are needed [124], the auxiliary target effect of which is hypertension [15]	←	poor prognosis in patients with gastric cancer [201], surgical removal of which leads to hypertension remission [12] mild increase of vascular inner diameter [200]	→
22	*PCDHGA1*	within human disease models using brain microvascular endothelial cells lacking a gamma-protocadherin gene: higher risks of blood-brain barrier dysfunction [125] comorbid with hypertension [126]	→	within the GEO-related meta-analysis: higher risks of breast cancer [127], which is also increasing with hypertension in obese, physically inactive, postmenopausal women and is reducible due to short-term aerobic activity [10]	→
23	*PDYN*	within human disease models using PDYN-knockout mice: reduced alcohol drinking [128] unlike alcohol addiction, which is comorbid with hypertension [129]	←	according to the database ClinVar report on SNP rs886056538, high risks of spinocerebellar ataxia [130], which can mark portal hypertension [131]	→
24	*PLA2G2D*	within human disease model using Pla2g2d-null mice: higher risks of psoriasis [132], which is comorbid with hypertension [133]	→	within human disease model using Pla2g2d-overexpressing transgenic mice: ameliorated psoriasis [132] comorbid with hypertension [133]	←
25	*PLA2G5*	within human disease models using Pla2g5-null mice: prevented lung endothelial dysfunction [134], which is a symptom of pulmonary hypertension [135]	←	within a cohort clinical study: poor prognosis in patients with gliomas [136], which can lead to intracranial hypertension [11]	→
26	*PLOD1*	within the PLOD1-null human lung cancer cell lines: reduced cancer cell proliferation [137] as well as anti-hypertensive drugs prevent lung cancer[138]	←	according to a transcriptome meta-analysis: high risks of gliomas [139], which can lead to intracranial hypertension [11]	→
27	*PPP1R3B*	within human disease models using Ppp1r3b-knockout mice: higher risks of fasting-induced hypoglycemia [140] comorbid with hypertension [141]	→	within human disease models using liver-specific overexpression Ppp1r3b mice: resistance to hypoglycemia [140] comorbid hypertension [141]	←
28	*PRLR*	within a herbal pharmaceutical report: *Withania somnifera* extract lows PRLR level as adaptogen against ageing-related diseases [142], eg, hypertension [143]	←	PRLR excess can characterize breast tumors [144], risks of which rises with hypertension in obese, physically inactive, postmenopausal women and is reducible due to short-term aerobic activity [10]	→

***Genes**: HTR2C,* 5-hydroxytryptamine receptor 2c; *KRT2,* keratin 2;; *LILRB3L,* leukocyte immunoglobulin-like receptor, subfamily b, member 3-like; *LYPD1,* ly6/plaur domain containing 1; *MORN1,* morn repeat containing 1; *MYOM2,* myomesin 2; *PCDHB9,* protocadherin beta 9; *PCDHGA1,* protocadherin gamma subfamily A1; *PDYN,* prodynorphin; *PLA2G2D,* phospholipase a2, group IID; *PLA2G5,* phospholipase a2, group V; *PLOD1,* procollagen-lysine, 2-oxoglutarate 5-dioxygenase 1;. ; *PPP1R3B,* protein phosphatase 1, regulatory subunit 3b; *PRLR,* prolactin receptor.

Table S3. *Cont*

No	Human Gene	Effect of the human gene expression alterations on the hypertension development [Ref]			
		**Downregulation (↓)**	HT	**Upregulation (↑)**	HT
**i**	**ii**	**iii**	**iv**	**v**	**vi**
29	*PYGL*	within a transcriptome study: increased survival in pancreatic ductal adenocarcinoma [145], treatment of which has hypertension among side effects [186]	←	within a cohort study: a poor prognosis in patients with gliomas [146], which can lead to intracranial hypertension [11]	←
30	*RBM3*	within human embryogenesis models using Rbm3-knockout mice: maternal cold stress causes abnormal brain development [147], which is non-invasive detectable due to the intracranial hypertension it causes [148]	→	in two cohort studies: higher risks of breast cancer [149], which increases with hypertension in obese, physically inactive, postmenopausal women and is reducible due to short-term aerobic activity [10]	→
31	*RETSAT*	within human diseases models using Retsat-knockout mice: autoimmunity development [150], a complication of which is hypertension [151]	→	in the pancreatic transcriptome of obese vs normal mice [152]: Retsat excess is DEG, while obesity and hypertension worsen metabolic syndrome [153]	→
32	*SLC16A12*	within a meta-analysis of cancer-related RNA-Seq data: poor prognosis in clear cell renal cell carcinoma [154], a risk-factor of which is hypertension [155]	→	within a cohort clinical study using luciferase reporter assay: higher risks of age-related cataract [156], which is comorbid with hypertension [157]	→
33	*SLC4A5*	within human disease models using Slc4a5-null mice: acidosis [158] comorbid with hypertension, both of which could be relieved due to plant-diets [159]	→	within a retrospective transcriptome meta-analysis: reduced risks of Alzheimer's disease [160], whereas hypertension is risk-factor of this disease [161]	←
34	*SMOC2*	within human disease models using Smoc2-knockout mice: relieved liver fibrosis [162], which is comorbid with portal hypertension [163]	←	within a cohort-based study: higher risks of gastric cancer [164], surgical removal of which leads to hypertension remission [12]	→
35	*SPINT1*	within human disease models using Spint1-deficient zebrafish: higher risks of skin melanoma [165] with brain metastases, while surgical resection of brain cancers relieves intracranial hypertension [166]	→	within a cohort-based clinical study: higher risks of liver fibrosis [167], which is comorbid with portal hypertension [163]	→
36	*SULF1*	within human disease models using Sulf1-knockout: higher risks of diabetic nephropathy [168], which is comorbid with hypertension [169]	→	within a retrospective transcriptome meta-analysis: poor prognosis in urothelial carcinoma [170], which is comorbid with hypertension [171]	→
37	*SYNC*	within human health models using syncoilin-null mouse: reduced physical activity [172], which is specific character of hypertensive patients [173]	→	within a cohort clinical study: higher risks of gastric cancer [174], surgical removal of which leads to hypertension remission [12]	→
38	*TC2N*	in a cohort study: poor outcome in breast cancer [175], risks of which rises with hypertension in obese, physically inactive, postmenopausal women and is reducible due to short-term aerobic activity [10]	→	within a retrospective transcriptome meta-analysis: poor prognosis in gastric cancer [176], surgical removal of which relieves hypertension [12]	→
39	*TECTA*	within human disease models using Tecta-knockout mice: higher risks of reduced hearing [177], a risk factor of which is hypertension too [178]]	→	within human disease models using mice injected with a tectorin fragment: autoimmune hearing loss [179], risks of which rises with hypertension [178]	→
40	*TMEM60*	within a retrospective transcriptome meta-analysis: both deficit and excess of TMEM60 are the reliable clinical markers of hypertension [180]	→	within a retrospective transcriptome meta-analysis: both deficit and excess of TMEM60 are the reliable clinical markers of hypertension [180]	→
41	*TXNRD2*	within human disease models using transgenic mice: heart contractile dysfunction [181], which rises risks of cardiac death in pulmonary hypertension [182]	→	within human disease models using pigs: Selenium-based diet rises Txnrd2 level and improves inflammatory status [183] as a care of cardiovascular health against the negative effects of hypertension [184]	←
42	*UCP2*	within human disease models using stroke-prone spontaneously hypertensive rats: Japanese style hypersodic diet causes Ucp2 deficit together with brain and kidney injury [185]	→	within human disease models using stroke-prone spontaneously hypertensive rats injected with a lentiviral vector carrying Ucp2 gene: Ucp2 excess prevents both brain and kidney injury at Japanese style hypersodic diet [185]	←

Genes: *PYGL*, glycogen phosphorylase l; *RBM3*, rna binding motif protein 3*RETSAT*, retinol saturase; *SLC16A12*, solute carrier family 16, member 12; *SLC4A5*, solute carrier family 4 member 5; *SMOC2*, sparc related modular calcium binding 2; *SPINT1*, serine peptidase inhibitor, kunitz type 1; *SULF1*, sulfatase 1; *SYNC*, syncoilin, intermediate filament protein; *TC2N*, tandem c2 domains, nuclear; *TECTA*, tectorin alpha; *TMEM60*, transmembrane protein 60; *TXNRD2*, thioredoxin reductase 2; *UCP2*, uncoupling protein 2.

S1. Supplementary methods for DNA sequence analysis

Two DNA sequence $S_{WT}=\{s_{WT;-90}\ldots s_{WT;i}\ldots s_{WT;-1}\}$ and $S_{MIN}=\{s_{MIN;-90}\ldots s_{MIN;i}\ldots s_{MIN;-1}\}$, which lengths are 70 bp that textually representing two variants of a given promoter located just in front of the transcription start site (TSS, $s_{WT;0}=s_{MUT;0}$; $s_i \in$ {a, c, g, t}) were the input data of our earlier created Web service SNP_TATA_Comparator (http://beehive.bionet.nsc.ru/cgi-bin/mgs/tatascan_fox/start.pl) [203] used, as shown within two textboxes "Basic sequence" and "Editable sequence," respectively, in Figure S2d (hereinafter, see the main section "2. Results").

At the beginning, upon each of these sequences $S\in\{S_{WT}, S_{MIN}\}$, an estimate "$-\ln(K_D(S))$" of the TATA-binding protein (TBP) binding affinity for the corresponding variant of this promoter was calculated, as:

$$-\ln(K_D) = 10.9 - 0.2\ [\ln(K_{SLIDE}\ K_{STOP}\ K_{BEND}), \quad \textbf{(1)}$$

where 10.9 (ln units) corresponds to the estimates of nonspecific TBP-DNA affinity (i.e., 10 mM [215]; 0.2 is the stoichiometric coefficient [213]; $-\ln(K_{STOP})$ as an empirical estimate of an affinity of the TBP for the most probable TBP-binding site according to Bucher's criterion [218] among all the possible 15 bp fragments of both DNA chains of the promoter treated, namely:

$$\boldsymbol{ln(K_{STOP})} = MAX\left\{\sum_{j=-1}^{13} w_{j;s_{i+j}}\right\}; \quad (2)$$

where w_{js} as an element of the Bucher's matrix [218], which corresponds to the case of the nucleotide s located within j-th position of the DNA sequence analyzed; MAX(ζ) is the highest ζ-value observed.

In Eq. (1), $-\ln(K_{SLIDE})$ is an empirical estimate of an affinity of the TBP for this promoter during TBP sliding along DNA in the ±5 bp local environment around the most probable TBP-binding site mentioned above that is heuristically calculated, as:

$$-\ln(K_{SLIDE}) = \textbf{MEAN}\ \{0.8[TA] + 3.4\mu + 35.1\}, \quad \textbf{(3)}$$

where [TA] is a weighted number of dinucleotide TA; μ as the arithmetical mean of the minor groove width of the DNA helix [216] of the TBP-binding site under consideration; 0.8, 3.4, and 35.1 are the linear regression coefficients optimized elsewhere [222]; MEAN(ζ) is arithmetic mean of all observed ζ-value.

In Eq. (1), $-\ln(K_{BEND})$ as an empirical estimate of an affinity of the TBP for the most probable TBP-binding site above said during allosteric rearrangement of the B-helical DNA of this site by bending its axis at right angles to fix the TBP-promoter complex that is calculated by the following formula:

$$-\ln(K_{BEND}) = \textbf{MEAN}\{0.9[TA, AA, TG, AG] + 2.5[TA, TC, TG] +14.4\}, \quad \textbf{(4)}$$

where 0.9, 2.5, and 14.4 are the linear regression coefficients as published elsewhere [222].

Next, the "$-\ln[K_D]$ values (Eqs. 1 - 4) are accompanied by their standard error of mean (SEM) estimated by means of all the possible nucleotide substitutions, $s_{\bullet;j}\rightarrow\xi$, at each position j within the above ±5 bp local environment around the most probable TBP-binding site, such as:

$$SEM(S_\bullet)=[(\Sigma_{1\leq i\leq 26}\Sigma_{\xi\ \{a,c,g,t\}}[\ln(K_D(\{s_{\bullet;i-13}\ldots\xi\ldots\bullet s_{\bullet;i+12}\})/K_D(\{s_{\bullet;i-13}\ldots s_{\bullet;i+j}\ldots s_{\bullet;i+12}\})^2)]]/(3*26)^{1/2} \quad \textbf{(5)}$$

As an intermediate result, there are two paired estimates "$-\ln(K_D(S_{WT})) \pm SEM(S_{WT})$" and "$-\ln(K_D(S_{MIN}))\pm SEM(S_{MIN})$" calculated upon the input sequences S_{WT} and S_{MIN} using Eqs. (1–5) that are statistically comparable with one another in the terms of Fisher's Z-test, namely:

$$Z = \textbf{abs}\ [\ln(K_{WT;D}\ /\ K_{MIN;D})]\ /\ [SEM(S_{WT})^2 + SEM(S_{MIN})^2]^{1/2}. \quad (6)$$

where Z as Fisher's Z-score as input for the corresponding procedure within the commonly accepted statistical package R [209], output of which is *p*-value of the probability rate of acceptance of the H_0-hypothesis "H_0: $K_D(S_{WT}) \neq K_D(S_{MIN})$".

Thereby, the final decision is made at its statistically significant level $\alpha < 0.05$ (where $\alpha = 1 - p$), as:

IF {*INEQUALITY* "$-\ln(K_{WT;D}) > -\ln(K_{MIN;D})$" is statistically significant},

THEN {*DECISION* is "S_{MIN} provides an underexpression of a given gene in comparison with S_{WT} as a norm"};

ELSE [**IF** {*INEQUALITY* "$-\ln(K_{WT;D}) < -\ln(K_{MIN;D})$" is statistically significant},

THEN {*DECISION* is "S_{MIN} provides an overexpression of a given gene in comparison with S_{WT} as a norm"},

OTHERWISE {*DECISION* is "alteration of the expression of this gene is insignificant"}.

Readers can see this DECISION in Figure S2d, namely: the text box "Result", the line "DECISION".

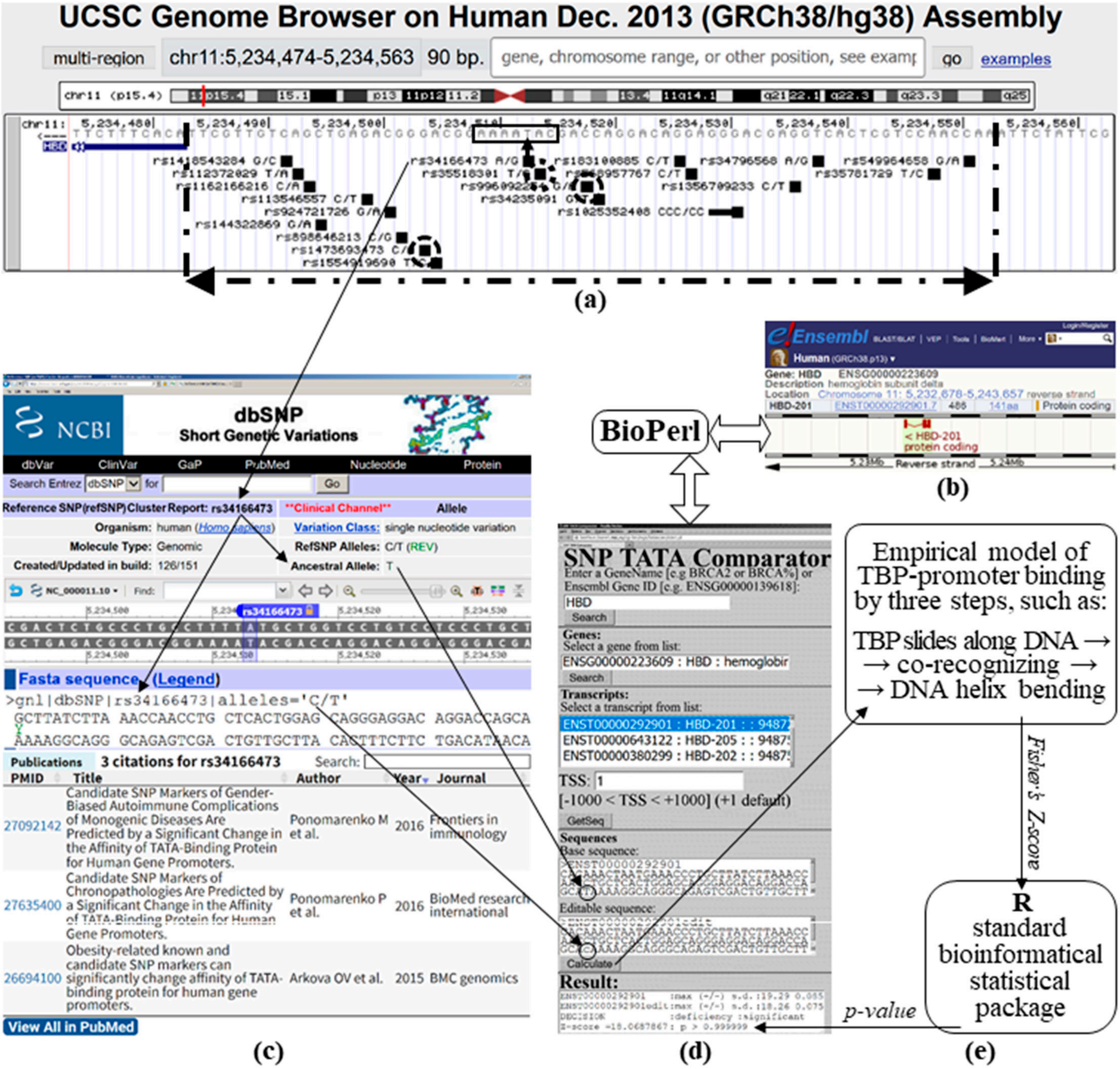

Figure S2. An illustrative example of the result produced by SNP_TATA_Comparator [203] in the case of the hypertension-related candidate SNP marker (rs34166473) reducing blood viscosity via *HBD* downregulation [199]. Legend: a) The UCSC Genome Browser [207] visualizes a 70 bp promoter (double-headed dash-and-dot arrow) of the human *HBD* gene in this example where there is only one TBP-binding site (framed) and SNP rs34166473 [211] with an arrow pointing to it; dotted circle: the only clinically proven SNP marker (rs35518301) of thalassemia together with malaria resistance within the human *HBD* promoter in question; dashed circle: the only candidate SNP marker (rs996092254) of HBD upregulation (and therefore a δ-hemoglobin excess) increasing the risk of hypertension, as one can see in Table 11; (**b**) the Ensembl database [210]. (**c**) The current build (No. 153) of the dbSNP database describes the SNP under study (rs34166473 in this example). (**d**) Our prediction of HBD deficiency (textbox "Result": row 3) caused by SNP rs34166473 (circled) by means of its both alleles (i.e., ancestral and minor ones as input data within two textboxes "Basic sequence" and "Editable sequence," respectively). Double-headed open arrows depict how our Web service SNP_TATA_Comparator [203] retrieved the ancestral DNA sequence of the human *HBD* promoter from the Ensembl database [210] using the Bioperl toolkit [208]. (**e**) Our bioinformatics model of the TBP–promoter binding in three steps, namely: (i) TBP slides along DNA ↔ (ii) TBP encounters a potential binding site for TBP (TBP-site) ↔ the B-form DNA of this TBP-site bends at right angles, which fixes the emerging TBP–promoter complex [213], as proven experimentally [214]. This model is based on the standard bioinformatical package of R [209]. Solid arrows: data flows when one predicts the manifestation of a given SNP using SNP_TATA_Comparator [203].

Table S4. Hypertension-related candidate SNP markers within the 70 bp proximal promoter of human genes *HBB*, *HBD*, and *PCDHB9* as calculated here.

#	*Human gene*	dbSNP ID rel. 153, [211]	DNA, genome sequence: 5′ flank	WT	min	3′ flank	K_D, nM: WT	min	Δ	Z	α	ρ	clinical data *or candidate SNP markers*	HT	Ref.
i	*ii*	iii	iv	v	vi	vii	viii	ix	x	xi	xii	xiii	xiv	xv	xvi
1		**rs35518301**	**caggaccagc**	**g**	**a**	**taaaaggcag**	**4**	**8**	**<**	**11**	$\mathbf{10^{-6}}$	**A**	**resistance to malaria and thalassemia**	←	**[212]**
2	*HBD*	rs34166473	aggaccagca	T	c	aaaaggcagg	4	12	<	18	10^{-6}	**A**	*low blood viscosity*	←	[199]
3		rs1473693473	ataaaaggca	G	t	ggcagagtcg	4	5	<	3	10^{-3}	**B**		←	
4		rs996092254	aggacaggac	C	t	agcataaaag	4	3	>	4	10^{-3}	B	*high-altitude environment provokes hyperhemo-globinemia & hypertension*	→	[103]
5		rs34500389	cagggctggg	C	t*	ataaaagtca	5	2	>	14	10^{-6}	A		→	
6		rs1160543272:<u>a</u>	ccagggctgg	G	<u>a</u>	cataaaagtc	5	4	>	3	10^{-2}	C		→	
7		rs1160543272:<u>t</u>	ccagggctgg	G	<u>t</u>	cataaaagtc	4.6	5.3	<	2	0.05	D	*low blood viscosity*	←	[199]
8		rs281864525	tgggcataaa	A	c*	gtcagggcag	5	7	<	7	10^{-6}	A		←	
9		rs63750953	ctgggcataa	AA	-	gtcagggcag	5	8	<	9	10^{-6}	A		←	
10	*HBB*	rs1554918265	gcataaaagt	C	t	agggcagagc	5	6	<	4	10^{-3}	B		←	
11		**rs33981098**	**agggctgggc**	**A**	**g, c**	**taaaagtcag**	**5**	**9**	**<**	**10**	$\mathbf{10^{-6}}$	**A**		←	
12		**rs33931746:g**	**gctgggcata**	**A**	**g**	**aagtcagggc**	**5**	**11**	**<**	**14**	$\mathbf{10^{-6}}$	**A**	**resistance to malaria and thalassemia**	←	**[212]**
13		**rs33931746:c**	**gctgggcata**	**A**	**c**	**aagtcagggc**	**5**	**8**	**<**	**9**	$\mathbf{10^{-6}}$	**A**			
14		**rs34598529**	**ggctgggcat**	**A**	**g**	**aaagtcaggg**	**5**	**18**	**<**	**24**	$\mathbf{10^{-6}}$	**A**		←	
15		**rs397509430**	**gggctgggca**	**T**	**-**	**aaaagtcagt**	**5**	**29**	**<**	**34**	$\mathbf{10^{-6}}$	**A**		←	
16		**rs33980857**	**gggctgggca**	**T**	**a, g, c**	**aaaagtcagt**	**5**	**21**	**<**	**27**	$\mathbf{10^{-6}}$	**A**		←	
17		rs1290084013	gaacaaaatt	G	t	accagaatgc	19	22	<	3	10^{-2}	C	mild increase of vascular inner diameter	←	[103]
18		rs1364916324	cggaggaaca	A	c	aattgaccag	19	24	<	4	10^{-3}	B		←	
19		rs1385825481	acggaggaac	A	g	aaattgacca	19	28	<	8	10^{-6}	A		←	
20		rs1015836045	agagacggag	G	a	aacaaaattg	19	22	<	3	10^{-2}	C		←	
21		rs934736711	gtccttgaca	A	g	aaaggaaaca	18	27	<	8	10^{-6}	A		←	
22	*PCDHB9*	rs1262387574	agtccttgac	A	g	aaaaggaaac	18	28	<	8	10^{-6}	A		←	
23		rs981373763	gaatgctacg	G	a*	AAGTCCTTGA	19	14	>	6	10^{-6}	A	*higher risk of gastric cancer surgical removal of which relieves hypertension*	→	[202, 12]
24		rs948650335	agaatgctac	G	a*	gaagtccttg	19	15	>	5	10^{-3}	B		→	
25		rs974379610	gaccagaatg	C	t	tacggaagtc	19	17	>	3	0.05	D		→	
26		rs962603908	tgaccagaat	G	a	ctacggaagt	19	9	>	12	10^{-6}	A		→	
27		rs141152529	CTGAAGAGAC	G	a*	GAGGAACAAA	19	17	>	3	0.05	D		→	

Notes. *Alleles*: wt, ancestral; min, minor; "–", deletion. K_D: dissociation constant of the TBP–DNA complex; $\alpha = 1 - p$: significance (the p value is given in Figure S2d); *Gene expression changes* (Δ): an increase (>) and decrease (<); Z: Fisher's Z-score; ρ: the heuristic rank of candidate SNP markers from the "best" (A) to the "worst" (E). *This SNP includes other neutral alleles.

S2. Supplementary methods for *in vitro* measurements

Recombinant full-length human TBP was expressed in *Escherichia coli* BL21 (DE3) cells transformed with the pAR3038-TBP plasmid (a kind gift from Prof. B. Pugh, Pennsylvania State University) by a previously described method [223] with two modifications: the IPTG concentration was 1.0 instead of 0.1 mM; the induction time was 3 instead of 1.5 h. For details of our protocol for production and purification of human TBP, see Ref. [224].

Eleven ODNs 26 bp in length listed in Table S1 were synthesized by the Biosynthesis Enterprise (Novosibirsk, Russia) and were purified by PAGE. Labeled double-stranded ODNs were prepared by ^{32}P labeling of both strands by means of T4 polynucleotide kinase (SibEnzyme, Novosibirsk) with subsequent annealing by heating to 95°C (at equimolar concentrations) and slow cooling (no less than 3 h) to room temperature. The duplexes were analyzed in a 15% nondenaturing polyacrylamide gel (1 × Tris-borate-EDTA buffer) and isolated by electroelution. For more details of our protocol for labeling of ODNs with ^{32}P, readers can see Ref. [224].

The equilibrium dissociation constants (K_D) were determined for the complexes of TBP with each 26-bp ODN in question. Experiments on association kinetics were conducted at four ODN concentrations (Figure S3a). The experiments with TBP–ODN binding were carried out at 25°C in binding buffer (20 mM 4-[2-hydroxyethyl-1-piperazineethanesulfonic acid [HEPES-KOH pH 7.6, 5 mM MgCl2, 70 mM KCl, 1 mM dithiothreitol (DTT), 100 μg/mL BSA, 0.01% of NP-40, and 5% of glycerol) at a fixed concentration (0.3 nM) of active TBP. The gels were dried, and Imaging Screen-K (Kodak, Rochester, NY, USA) was exposed to these gels for analysis on a Molecular Imager PharosFX Plus phosphorimager (Bio-Rad, Herts, UK). The resulting autoradiographs were quantitated in the Quantity One 4.5.0 software (Bio-Rad) as displayed in Figure S3b. Using these data as input for publicly available software Graph-Pad Prism 5 (http://graphpad-prism.software.informer.com/5.01), we calculated the equilibrium dissociation constant (K_D). For more details of our protocol for *in vitro* measurements of the equilibrium dissociation constant for TBP–ODN complexes, one can see Ref. [225].

Table S5. ODNs identical to fragments of ancestral (WT) and minor (SNP ID) variants of the human gene promoters studied in this work

#	*Gene*	WT or SNP ID	Sequence, 26 bp, direct, 5′→3′	#	*Gene*	WT or SNP ID	Sequence, 26 bp, direct, 5′→3′
1	*HBD*	WT	acaggaccagcataaaaggcagggca	4	*HBB*	WT	cagggctgggcataaaagtcagggca
2	*HBD*	rs35518301:G	acaggaccagcGtaaaaggcagggca	5	*HBB*	rs34500389:T	cagggctgggTataaaagtcagggca
3	*HBD*	rs34166473:C	acaggaccagcaCaaaaggcagggca	6	*HBB*	rs33980857:A	cagggctgggcaAaaaagtcagggca
				7	*HBB*	rs34598529:G	cagggctgggcatGaaagtcagggca
				8	*HBB*	rs33931746:G	cagggctgggcataGaagtcagggca
				9	*HBB*	rs33931746:C	cagggctgggcataCaagtcagggca
				10	*HBB*	rs281864525:C	cagggctgggcataaaCgtcagggca
				11	*HBB*	rs63750953:Δ	cagggctgggcataagtcagggcaga

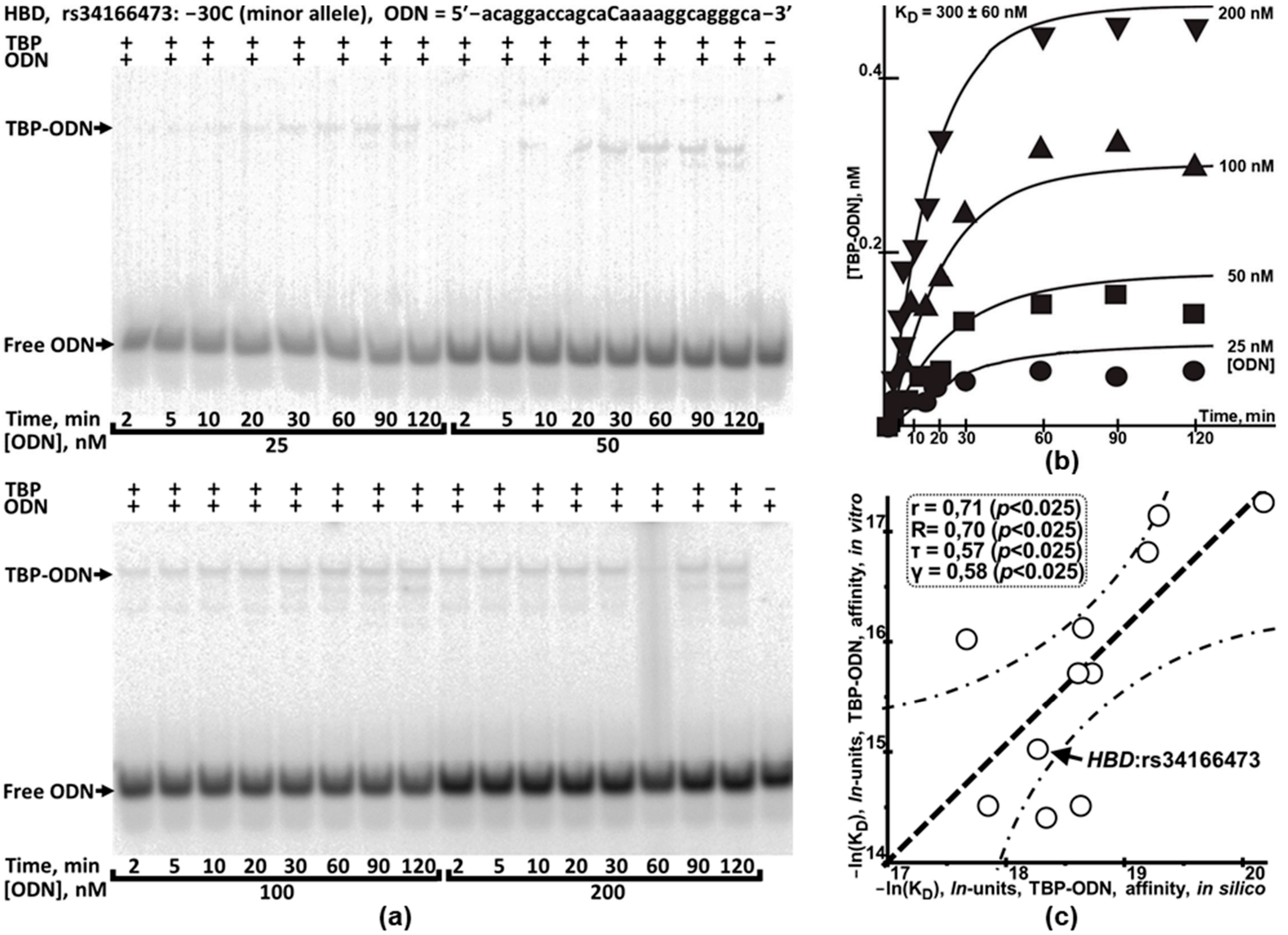

Figure S3. Statistically significant correlations between *in silico*–predicted and *in vitro*–measured values of equilibrium dissociation constant K_D of TBP–promoter affinity expressed in "moles per liter" units converted to a natural logarithm scale, ln units. *Legend*: see legends of Figures S1. (a) Electropherograms for the minor allele of SNP rs1473693473 (in the human *HBD* gene promoter) as a vivid example. The concentration of TBP was 0.3 nM in all the experiments. Concentrations of 26 bp oligodeoxyribonucleotides (ODNs) are indicated. (b) Dependences of reaction rates on ODN concentrations in cases of minor alleles of the examined SNP. K_D was inferred from these dependences serving as input data for publicly available software GraphPad Prism 5 (http://graphpad-prism.software.informer.com/5.01). (c) The tested correlations.

© 2022 by the authors. Submitted for possible open access publication under the terms and conditions of the Creative Commons Attribution (CC BY) license (http://creativecommons.org/licenses/by/4.0/).
